# Releasable and traceless PEGylation of arginine-rich antimicrobial peptides†
†Electronic supplementary information (ESI) available: Experimental procedures, synthesis and characterization of rPEGylation reagents and peptide–PEG conjugates, kinetic studies of PEGylation and dePEGylation (HPLC) and controls are included. See DOI: 10.1039/c7sc00770a


**DOI:** 10.1039/c7sc00770a

**Published:** 2017-03-30

**Authors:** Y. Gong, D. Andina, S. Nahar, J.-C. Leroux, M. A. Gauthier

**Affiliations:** a Institute of Pharmaceutical Sciences , Department of Chemistry and Applied Biosciences , Swiss Federal Institute of Technology Zurich (ETHZ) , Zurich 8093 , Switzerland; b EMT Research Center , Institut National de la Recherche Scientifique (INRS) , Varennes J3X 1S2 , Canada . Email: gauthier@emt.inrs.ca

## Abstract

Arginine-rich antimicrobial peptides (AMPs) are emerging therapeutics of interest. However, their applicability is limited by their short circulation half-life, caused in part by their small size and digestion by blood proteases. This study reports a strategy to temporarily mask arginine residues within AMPs with methoxy poly(ethylene glycol). Based on the reagent used, release of AMPs occurred in hours to days in a completely traceless fashion. *In vitro*, conjugates were insensitive to serum proteases, and released native AMP with full *in vitro* bioactivity. This strategy is thus highly relevant and should be adaptable to the entire family of arginine-rich AMPs. It may potentially be used to improve AMP-therapies by providing a more steady concentration of AMP in the blood after a single injection, avoiding toxic effects at high AMP doses, and reducing the number of doses required over the treatment duration.

## Abstract 2

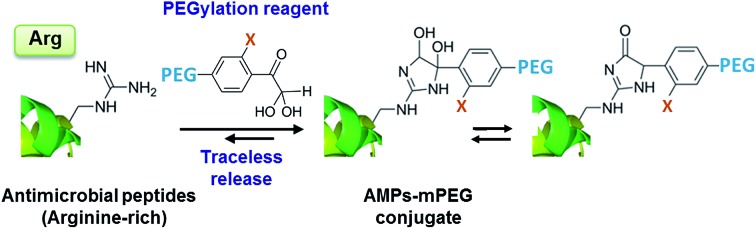
This study reports a strategy to temporarily mask arginine residues within antimicrobial peptides (AMPs) with methoxy poly(ethylene glycol) (mPEG). PEGylation protects AMPs from serum proteases, and can be released at a pharmaceutically-relevant rate. Fully active and unmodified (*i.e.*, native) AMPs are released with time.

## Introduction

Multi-drug resistance is a major challenge in the treatment of bacterial infections, because resistant strains respond poorly to conventional antibiotics. This points to the need for alternative therapeutic agents, such as antimicrobial peptides (AMPs). Natural AMPs protect organisms against microbes by exerting a direct antibiotic activity, in addition to acting as effectors and regulators of the innate immune system.^1^–^3^ Synthetic variants are in development or in clinical evaluation.^3^ As pharmaceuticals, however, AMPs are small and have short circulatory half-lives, which can be on the order of tens of minutes,^4^ and are susceptible to proteolysis. Indeed, an important subset of AMPs are rich in arginine, which is necessary for interaction with bacterial membranes,^4^ but, unfortunately, also the target of trypsin-like proteases. These combined challenges can impose higher or more frequent dosing to maintain adequate blood levels, which could be incompatible with the toxic side-effects observed at high doses, especially for lytic AMPs.^1^

**Fig. 1:**
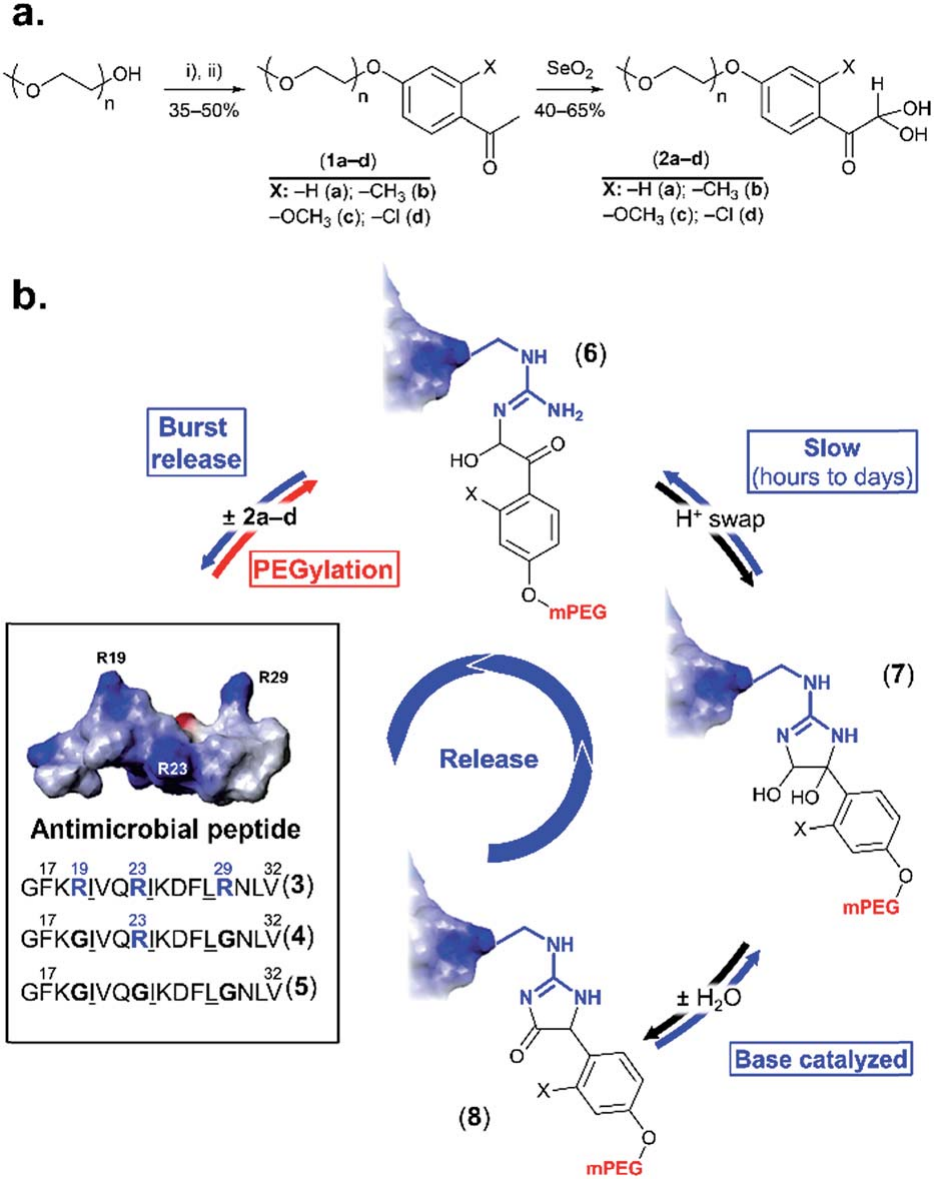
rPEGylation of arginine-rich AMPs. (a) Synthesis of arginine-reactive polymers ((i): tosyl chloride, triethylamine; (ii): *o*-substituted *p*-hydroxyacetophenone, K_2_CO_3_). (b) Sequence and 3D structure (inset from ref. 21 with permission) of model AMP (**3**) and controls with one (**4**) or no arginine residues (**5**). Structure of three forms (**6–8**) of the conjugates obtained. Letters represent single letter codes for amino acids (underlined are d-amino acids).

Established strategies for addressing such shortcomings, such as PEGylation (grafting of methoxy poly(ethylene glycol) (mPEG)),^5^–^8^ are generally inapplicable because they can inactivate the small AMP. However, the emerging concept of releasable PEGylation (rPEGylation)^7^,^9^–^12^ could provide the solution, as “native” (fully active) AMPs would be released in the body. Nollmann *et al.*^13^ have recently reported the rPEGylation of proline-rich AMPs by their N-terminal extension with mPEG and a linker that can be digested by serum proteases. While promising, current rPEGylation chemistry^12^ almost exclusively involves protecting lysines or the N-terminus of peptides/proteins. In addition, strategies that do not rely on blood-borne triggers (enzymes), which might display patient-variability, could be more reproducible *in vivo*. Developing rPEGylation chemistry to temporarily mask other residues is therefore timely and of interest. This study demonstrates the traceless rPEGylation of arginine residues, a major recurrent amino acid within the family of arginine-rich AMPs, using rPEGylation agents bearing phenylglyoxal units (Fig. 1a).

Phenylglyoxal has been used for studying the function, local polarity, *etc.*, of arginine (and citrulline) residues within proteins.^14^–^16^ In contrast to aliphatic glyoxals that permanently modify arginine,^17^,^18^ resonance of the ketone with the aromatic ring lowers the stability of the arginine–phenylglyoxal adduct, making this a possible route for rPEGylation.^19^,^20^ As a model, a short AMP containing three arginine residues (**3** in Fig. 1b) derived from LL-37,^21^ was selected. LL-37 fragment derivatives have been considered for chronic otitis media.^22^ Variants containing one (**4**) or no arginine residues (**5**) served as controls.

## Results and discussion

**Fig. 2:**
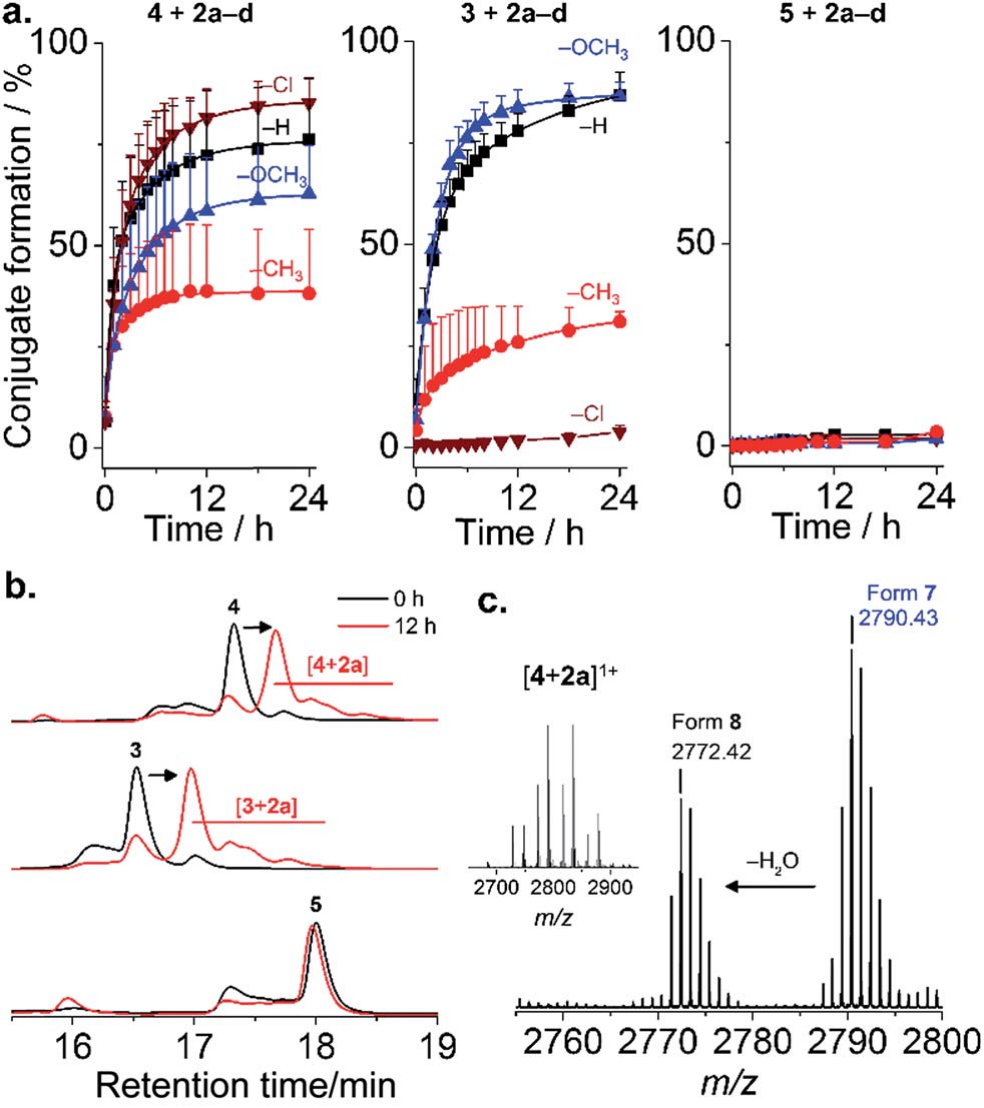
PEGylation of AMPs. (a) Kinetics of reaction of **3–5** with **2a–d** in phosphate buffer pH 7.4. Mean + SD (*n* = 3). (Conjugate formation = 100 × (1 – fraction residual AMP)); (b) representative chromatograms; (c) mass spectrum of [**4** + **2a**] ([M + H]^+^ calcd 2790.43 (found: 2790.43); [M – H_2_O + H]^+^ calcd 2772.42 (found: 2772.42)).

Four mPEG–phenylglyoxal reagents (**2a–d**) bearing substituents ortho to the glyoxal were prepared (Fig. 1a and S1–15†). A short mPEG (350 g mol^–1^) was initially chosen to facilitate analysis of the conjugates by mass spectrometry. PEGylation using **2a–d** was first analyzed with AMP **4** as it contains only a single arginine. Conjugation with a small excess (4 eq.) of **2a–d** proceeded smoothly over 24 h at pH 7.4 (Fig. 2a and S16–19†). The disappearance of **4** was accompanied by the appearance of peaks for the mono-PEGylated conjugate, which were collectively isolated and identified by mass spectrometry (Fig. 2b and c). As depicted in Fig. 1b, **2a–d** first transiently formed a hemiaminal (**6**), which then more slowly cyclized to the dihemiaminal (**7**). Dehydration of **7** produced form **8**, which was observed for **2a–c**, but not for **2d**. PEGylation of AMP **3** (three arginine residues) proceeded smoothly (Fig. 2a and b and S20–23†), yielding 1.5, 1.3, and 2.0 mPEG units per peptide after 24 h for **2a**, **2b**, and **2c**, respectively (**2d**, *vide infra*). PEGylation was selective to arginine residues, as no reaction of **2a–d** occurred with **5** or two additional control peptides with multiple nucleophilic residues (Fig. 2, S24 and 25†). Thus, because glyoxals react with thiols (Fig. S26†), **2a–d** can be considered as arginine-specific PEGylation agents for thiol-free peptides.

The rate of reaction of **4** with **2a–d** was influenced by substituents on the aromatic ring. Baburaj *et al.*^23^ have shown that a methoxy group para to the glyoxal decreases its reactivity towards arginine. A similar effect is therefore expected for mPEG, in addition to steric effects. Indeed, increasing the molecular weight of **2a** using mPEG 1 or 2 kDa decreased the conversion achieved within 24 h by a factor of ∼2 (only small differences between the two longer mPEGs; Fig. S27†). Of course, PEGylation can be accelerated by increasing the amount of **2a–d** used beyond 4 eq., or by using carbonate buffers that catalyze coupling.^23^ Predicting “ortho-effects” for the other substituents is complicated due to hindrance and potential interaction with the glyoxal. Reactivity can be ranked as Cl (fastest) > H > OCH_3_ > CH_3_, though was similar for **2a**, **2c**, and **2d**. This suggests that the electron-donating properties of CH_3_ and OCH_3_ reduce reactivity, while the electron-withdrawing properties of Cl accelerate the reaction. A similar trend was observed for AMP **3**, with the exception of **2d** (Cl), which did not react. This intriguing result suggested some influence of AMP sequence. Indeed, it is known that while proteins feature many exposed arginine residues, invariably only few react.^24^,^25^

**Fig. 3:**
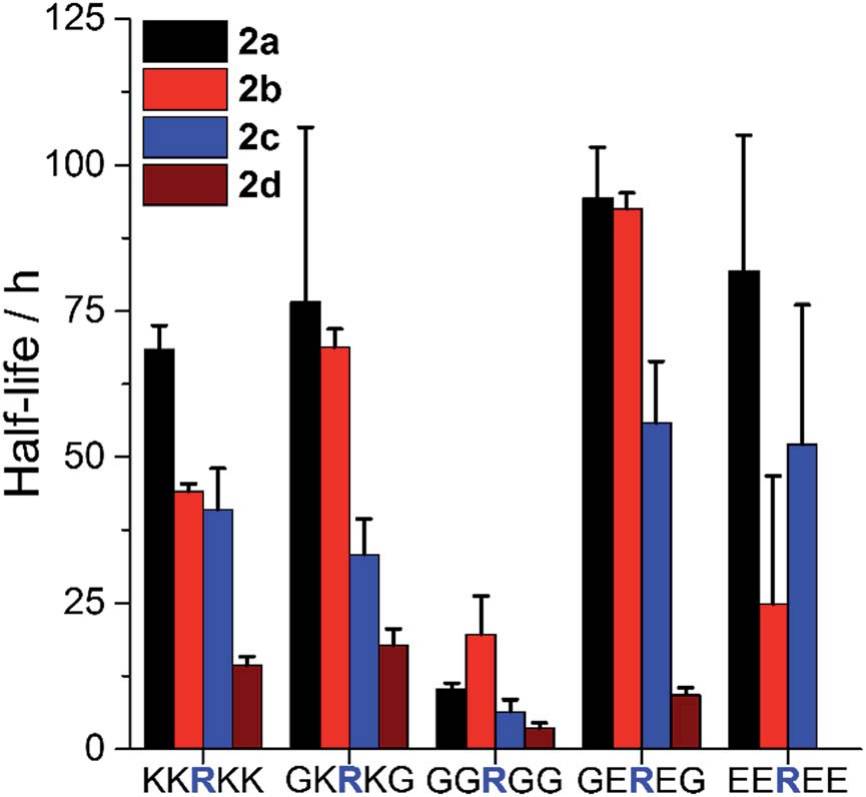
Influence of AMP sequence on PEGylation kinetics. Half-life of reaction of peptides with 1.5 eq. **2a–d** at pH 7.4 to assess the influence of steric hindrance and charge on reactivity. Mean + SD (*n* = 3). Full ANOVA in Table S1.†

To examine such sequence effects for AMPs, the PEGylation of short peptides containing arginine and variable flanking residues was evaluated (Fig. 3, full ANOVA in Table S1†). **2a–d** reacted fastest when the arginine was not sterically hindered (neighbors were glycine, G) and slower when flanked by bulkier lysine (K) or glutamic acid (E). The charge of the microenvironment influenced the reactivity. When the first neighboring residues were negatively charged, peptides reacted more slowly than for correspondingly positively charged ones for **2a** (H) and **2c** (OCH_3_) but the opposite was observed for **2d** (Cl). **2a** was insensitive to the nature of the second neighboring residue. The influence of the second neighbor on the reactivity of **2b** (CH_3_) and **2c** (OCH_3_) was less predictable, and **2d** (Cl) did not react at all with the peptide bearing four negative charges. Thus, even though PEGylation is affected by AMP sequence, **2a** appeared to be the least affected by the local charge and may be the most predictable PEGylation agent.

**Fig. 4:**
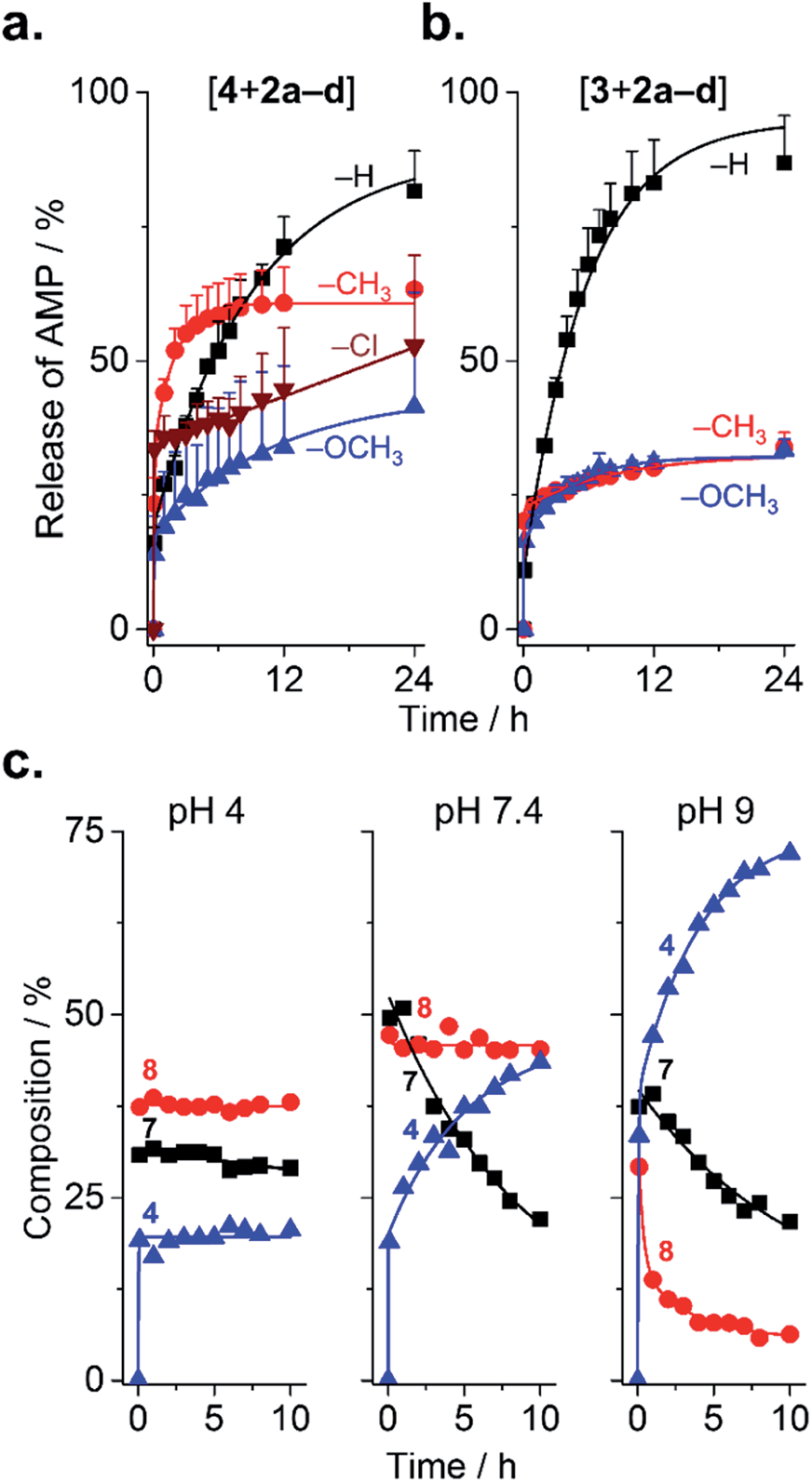
Release of AMPs from AMP–mPEG conjugates. (a and b) Influence of substituents on the release of peptide from conjugates of **4** or **3** with **2a–d**. Mean + SD (*n* = 3). (c) Effect of pH on the release of **4** from [**4** + **2c**] conjugates (*n* = 1).

Because substituents influenced the rate of PEGylation, it stands to reason that they should also influence de-PEGylation, though this has never been investigated. As PEGylation was performed in phosphate buffer (*vs.* borate buffer, *vide infra*), conjugates were present as a combination of forms **6–8** (Fig. 1b). This enables us to assess de-PEGylation from all of these species, for a more general view of rPEGylation using phenylglyoxals. Release of the native peptide was triggered by dilution to 0.2 mg mL^–1^ in buffer containing 10% serum and monitored by HPLC (Fig. 4). All profiles displayed burst release within minutes due to hydrolysis of form **6** of the conjugates (Fig. 4a and b). Burst release was similar for all conjugates (∼11–23%), reflecting the relative proportion of form **6** present at time zero. Thereafter, different release profiles were observed: for conjugates of **2a**, both **3** and **4** were released almost completely over 24 h (82% for **4** and 87% for **3**). Because [**4** + **2a**] contained both forms **7** and **8**, this result indicates that all steps from **8** to **4** in Fig. 1b were possible. Release of **4** from [**4** + **2d**] occurred in a sustained, even linear manner, to reach ∼50% over 24 h. In contrast, conjugates of **2b** (CH_3_) and **2c** (OCH_3_) displayed similar release profiles, which were incomplete and plateaued. This result prompted a more detailed analysis, which was possible because forms **7** and **8** of [**4** + **2c**] were distinguishable by HPLC. At near neutral pH, form **7** progressively released **4** with a half-life of ∼10 h, while form **8** was stable (Fig. 4c). Acidic pH stabilized all forms of the conjugate, while alkaline pH accelerated release. These results indicate the substituents on **2b** and **2c** prevented hydration from **8** → **7** at neutral pH, resulting in the plateaus in Fig. 4a and b. Thus, in general, isolating form **7** of the conjugates would be desirable to avoid burst-(form **6**) or incomplete release (form **8**) of AMP at physiological pH. Fortunately, form **7** can be selectively and conveniently prepared using borate buffer because it accelerates the reaction from **6** → **7** and complexes the 1,2-diol of **7**, which prevents dehydration.^18^,^20^,^23^ This was evidenced by mass spectrometry for [**4** + **2a–d**] prepared in borate, and by the lack of burst release even after storage (dry, –20 °C) for 1 week (Fig. S28 and 29†).

The mechanism of de-PEGylation presumably occurs *via* sequential nucleophilic addition of water, in agreement with the observed pH dependence. Analysis at lower and higher serum content (0–50%) had no effect on release (Fig. S30†), suggesting again that water and not serum nucleophiles are responsible for release. This is also consistent with mPEG molecular weight only slightly affecting release, within the range tested (0.35–2 kDa; Fig. S27†). While a detailed analysis is complex, the apparent rates of de-PEGylation from form **7** of the conjugates obtained by non-linear regression (Table S2†) ranked as Cl (slowest) > H = OCH_3_ > CH_3_, which is consistent with the ranking found for PEGylation.

In order to validate that the PEGylated AMPs are resistant to proteolysis, [**3** + **2a**] (2 kDa) was incubated in ∼90% human serum (final concentration) spiked with 0.01 wt% trypsin for up to 24 h. To prevent de-PEGylation during this incubation period (*i.e.*, to test the protease-resistance of the conjugate rather than the released AMP (known to be degradable; *vide infra*)), the concentration of [**3** + **2a**] was maintained high, and a small excess of free **2a** was kept in solution. Based on Fig. 2 and 4, these conditions promote the forward PEGylation reaction and disfavor de-PEGylation. Following the incubation period (1 h, 6 h, or 24 h), serum proteins and trypsin were removed to stop proteolysis, and the integrity of the AMP **3** within the conjugate was evaluated *via* its anti-proliferative effect on *E. coli*. As seen in Table 1, the native AMP **3** rapidly lost its antimicrobial properties in less than 6 h of incubation in trypsin-spiked serum, with partial loss of activity already evident even after 1 h. In contrast, not only was the IC_50_ of [**3** + **2a**] not statistically different from that of the native control AMP **3**, no change in this value was observed even after a 24 h incubation in trypsin-spiked serum. These results confirm that de-PEGylation of the conjugate did not occur in the conditions selected for serum-exposure, and that the PEGylated AMP was entirely resistant to proteolysis, for at least 24 h.

**Table 1:** Antibacterial properties (IC_50_) of **3** and [**3** + **2a**, **c**] bio-conjugates determined after incubation in serum for pre-defined times (and removal of serum proteins). Mean ± SD (*n* = 3–4)

Serum exposure^*a*^	IC_50_ (μM)			
	Control (6 h, no serum)	1 h	6 h	24 h
**3**	8 ± 5^*b*^	40 ± 30^*d*^	n.a.^*c*^	n.a.
[**3** + **2a**]	12 ± 7	9 ± 5	8 ± 4	8 ± 5
[**3** + **2c**]	40 ± 20^*d*^	20 ± 10	20 ± 10	50 ± 20^*d*^

^*a*^Incubation was performed under conditions that favour the forward PEGylation reaction.

^*b*^Fresh solution (0 h).

^*c*^n.a.: No antibacterial effect was observed.

^*d*^Statistically significant difference (ANOVA, Tukey, *p* = 0.05) *vs.* control **3** (not exposed to serum).

Considering that the arginine residues of arginine-rich AMPs are essential for exerting their anti-bacterial properties,^4^ the PEGylated AMPs herein are, *a priori*, not expected to be bioactive. Nevertheless, because of this eventuality, and because a bioactive mPEG–AMP conjugate may differ in its selectivity towards bacterial *vs.* eukaryotic cells, this parameter must be evaluated. Unfortunately, a permanently PEGylated AMP reproducing the chemical features of the mPEG–AMP conjugates herein (*i.e.*, modification of arginine residues; aromatic groups at conjugation site) are not readily available. A permanently PEGylated AMP is necessary because the high dilution and extended duration of the anti-proliferation assay (16 h at 37 °C) is expected to promote maximum release of AMP **3** from the rPEGylated conjugates (Fig. 4). Therefore to address this, [**3** + **2c**] was selected for analysis because **2c** reacts with arginine residues similarly to **2a** (Fig. 2a), yet only partially de-PEGylates (Fig. 4b). Compared to [**3** + **2a**], a ∼2.5–6-fold higher IC_50_ was observed for [**3** + **2c**] in Table 1, which favorably correlates with the expected partial de-PEGylation of the bio-conjugate (∼30% in Fig. 4b). Considering that the mPEG–AMP conjugates are resistant to proteases, this increase of IC_50_ was attributed to the lack of bio-activity of the PEGylated AMP. Of course, the validity of this statement should be tested on an AMP-to-AMP basis, because of the variability of sequence and the position of the arginine within it.

As a final consideration, upon release from the bio-conjugate, **2a–d** are expected to be eliminated from the body either as-is, or coupled to endogenous nucleophiles (Fig. S26† for cysteine). It is therefore noteworthy to mention that the *in vitro* IC_50_ values of **2a–d** towards hepatocytes were in the mM range (Fig. S31†), which is *ca.* 2–3 orders of magnitude higher than the IC_50_ of AMP **3**, as well as the blood concentrations initially expected (∼μM) after administration of typical doses of AMPs (∼mg kg^–1^).^26^ Of course, toxicity should be evaluated *in vivo* and will depend on the dosing regimen.

## Conclusions

This study presents substituted phenylglyoxal mPEGs that selectively PEGylate arginine in AMPs, protect them from serum proteases, and release them in a traceless fashion with full bioactivity at a rate influenced by the substituent. From a therapeutic standpoint, the most desirable rate of release for a given AMP will depend on its identity, biodistribution, and dose. Extending the circulation time of AMPs from tens of minutes to *ca.* several hours to 1–2 days may potentially improve the treatment of bacterial infections by maintaining an effective concentration over this period in the blood. This strategy, however, may be inappropriate should longer circulation/release times be desired. This chemistry should be adaptable to the entire family of arginine-rich AMPs, and those containing multiple arginine residues could even be selectively PEGylated using protecting groups during synthesis. Finally, the catalytic properties of buffers such as borate or carbonate, which can be used to create more complex branch-like bio-conjugates (by multiple PEGylation of a single arginine residue), also offer exciting new opportunities for bio-conjugate design.

## Supplementary Material

Supplementary information

## References

[cit1] Hancock R. E. W., Sahl H.-G. (2006). Nat. Biotechnol..

[cit2] Boman H. G. (2003). J. Intern. Med..

[cit3] Fjell C. D., Hiss J. A., Hancock R. E. W., Schneider G. (2012). Nat. Rev. Drug Discovery.

[cit4] Chan D. I., Prenner E. J., Vogel H. J. (2006). Biochim. Biophys. Acta.

[cit5] Veronese F. M. (2001). Biomaterials.

[cit6] Roberts M. J., Bentley M. D., Harris J. M. (2002). Adv. Drug Delivery Rev..

[cit7] Pasut G., Veronese F. M. (2012). J. Controlled Release.

[cit8] Pasut G., Veronese F. M. (2007). Prog. Polym. Sci..

[cit9] Greenwald R. B., Choe Y. H., McGuire J., Conover C. D. (2003). Adv. Drug Delivery Rev..

[cit10] Filpula D., Zhao H. (2008). Adv. Drug Delivery Rev..

[cit11] Shechter Y., Mironchik M., Saul A., Gershonov E., Precido-Patt L., Sasson K., Tsubery H., Mester B., Kapitkovsky A., Rubinraut S., Vachutinski Y., Fridkin G., Fridkin M. (2007). Int. J. Pept. Res. Ther..

[cit12] Gong Y., Leroux J.-C., Gauthier M. A. (2015). Bioconjugate Chem..

[cit13] Nollmann F. I., Goldbach T., Berthold N., Hoffmann R. (2013). Angew. Chem., Int. Ed..

[cit14] Bicker K. L., Subramanian V., Chumanevich A. A., Hofseth L. J., Thompson P. R. (2012). J. Am. Chem. Soc..

[cit15] Stipani I., Mangiullo G., Stipani V., Daddabbo L., Natuzzi D., Palmieri F. (1996). Arch. Biochem. Biophys..

[cit16] Wang S., Wang X., Shi W., Wang K., Ma H. (2008). Biochim. Biophys. Acta, Proteins Proteomics.

[cit17] Gauthier M. A., Ayer M., Kowal J., Wurm F. R., Klok H.-A. (2011). Polym. Chem..

[cit18] Gauthier M. A., Klok H.-A. (2011). Biomacromolecules.

[cit19] Takahashi K. (1968). J. Biol. Chem..

[cit20] Baburaj K., Saeed A., Azam N., Durani S. (1991). Biochim. Biophys. Acta.

[cit21] Li X., Li Y., Han H., Miller D. W., Wang G. (2006). J. Am. Chem. Soc..

[cit22] Fox J. L. (2013). Nat. Biotechnol..

[cit23] Baburaj K., Durani S. (1991). Bioorg. Chem..

[cit24] Suckau D., Mak M., Przybylski M. (1992). Proc. Natl. Acad. Sci. U. S. A..

[cit25] Patthy L., Thész J. (1980). Eur. J. Biochem..

[cit26] Fukumoto K., Nagaoka I., Yamataka A., Kobayashi H., Yanai T., Kato Y., Miyano T. (2005). Pediatr. Surg. Int..

