# Supplementary material for: Releasable and traceless PEGylation of arginine-rich antimicrobial peptides
Source: Chem Sci. 2017 Mar 30;8(5):4082–6. doi: 10.1039/c7sc00770a (PMC6094173; doi:10.1039/c7sc00770a)
Supplement: Supplementary file 1 [file SC-008-C7SC00770A-s001.pdf]

Electronic Supplementary Material (ESI) for Chemical Science.
This journal is © The Royal Society of Chemistry 2017

Supporting information for

Releasable and Traceless PEGylation of Arginine-rich Antimicrobial Peptides

Yuhui Gong[1], Diana Andina[1], Sharifun Nahar,[2] Jean-Christophe Leroux[1], and Marc A Gauthier[2,*]

[1] Swiss Federal Institute of Technology Zurich (ETHZ), Department of Chemistry and Applied Biosciences, Institute of Pharmaceutical Sciences, Vladimir-Prelog-Weg 1–5/10, 8093 Zurich, Switzerland.

[2] Institut National de la Recherche Scientifique (INRS), EMT Research Centre, 1650 boul. Lionel-Boulet, Varennes, J3X 1S2, Qc, Canada

* Corresponding author email: gauthier@emt.inrs.ca

Experimental section

1. Materials

4-Hydroxyacetophenone, poly(ethylene glycol) 350 monomethyl ether (mPEG), silica gel (60 Å, 230–400 mesh), and *p*-toluenesulfonyl chloride were obtained from Fluka (various global suppliers). Fetal bovine serum, hydrochloric acid (37%), 4'-hydroxy-2'-methylacetophenone, selenium dioxide, triethylamine, and trifluoroacetic acid were obtained from Sigma-Aldrich (various global suppliers). 4'-Hydroxy-2'-methoxyacetophenone was obtained from Apollo Scientific Limited (Cheshire, UK). 4'-Hydroxy-2'-chloroacetophenone was obtained from AK Scientific, Inc. (Union City, CA, USA). H_2O_2 (30 wt%) was obtained from Merck (Darmstadt, Germany). In addition to the chemicals above, all buffer salts and solvents were purchased as the highest possible grade, and used as received. Deuterium oxide and chloroform-d were purchased from Cambridge Isotope Laboratories, Inc. (Tewksbury, MA, USA). Peptides (from N- to C-terminus) GGRGGW, GEREGW, EEREEW, GKRKGW, KKRKKW, GKGKGW, and GHGSGYG were purchased N-terminally acetylated and C-terminally amidated from Selleck Chemicals (Houston, TX, USA). Fluorescent peptides (from N- to C-terminus) (FITC)-GFKR{D-I}VQR{D-I}KDF{D-L}RNLV (**3**), (FITC)-GFKG{D-I}VQR{D-I}KDF{D-L}GNLV (**4**), and (FITC)-GFKG{D-I}VQG{D-I}KDF{D-L}GNLV (**5**) were purchased C-terminally amidated from Chempeptide Limited (Shanghai, PR China). Note that for efficient N-terminal labeling, a seven-atom aminohexanoyl spacer (NH_2-CH_2-CH_2-CH_2-CH_2-CH_2-COOH) is inserted between the fluorophore (fluoroscein) and the N-terminus of the peptide. All peptides were purchased at >95% purity and were supplied with analytical chromatograms and mass spectra. HepG2 cells were bought from ATTC (Manassas, VA, USA) and used from passage number 3 to 14. Minimum Essential Medium (MEM) containing GlutaMAX™, MEM without Phenol Red or glutamine, GlutaMAX™, fetal bovine serum (FBS), phosphate buffered saline (PBS), Penicillin-Streptomycin (10,000 $U \cdot mL^{-1}$) and 0.25 % Trypsin/ethylenediaminetetraacetic acid (EDTA) were obtained from Invitrogen (ThermoFisher Scientific, Zug, CH). CellTiter 96® AQ_{ueous} One Solution Cell Proliferation Assay (MTS) was purchased from Promega Corporation (Madison, WI, USA).

2. Analysis

NMR spectra were acquired on a Bruker AV400 spectrometer (Bruker BioSpin, Fällanden, Switzerland) operating at 400 MHz for protons. Chemical shifts (δ) are reported in ppm relative to residual solvent signals. Semi-preparative and analytical chromatography (for purification and monitoring kinetics, respectively) were performed with a high-performance liquid chromatography (HPLC) system (Merck HITACHI, Tokyo, Japan) equipped with an L-7100 pump, a Diode Array L-7455 detector and an FL L-7485 fluorescence detector [$\lambda_{ex/em}$ = 488 nm/525 nm]. The XBridge™ BEH300 Prep C18 column (5 μm, 10 mm × 150 mm) was used for purification and XBridge™ C18 column (5 μm, 4.6 mm × 250 mm) was used for monitoring the kinetic process. The mobile phase was water and acetonitrile (ACN), both supplemented with 0.1 vol% trifluoroacetic acid (TFA), and the flow rate was 4.4 mL/min for purification and 1.0 mL/min for kinetic measurements, respectively. A gradient method (0–2 min 0% ACN; 25 min 100% ACN; 30 min 0% ACN was used for both purification and kinetic studies. Liquid chromatography-mass spectrometry (LC-MS) was performed using a Rheos Allegro quaternary pump, Accela™ PDA Detector, XBridge C18 column (5 μm, 4.6 mm × 250 mm) and a Thermo LTQ XL mass spectrometer (Thermo Fisher Scientific, Vernon Hills, IL, USA). Mobile phases and gradients were the same as above. Data was analyzed with XCalibur control software. Matrix-assisted laser desorption/ionization time-of-flight (MALDI-TOF) MS was performed using a Bruker UltraFlex II spectrometer. An Infinite M200® Tecan microplate reader (Männedorf, Switzerland) was used to record UV absorbance and fluorescence [UV: 452 nm;

FL: $\lambda_{ex/em}$ =312 nm/395 nm] of the conjugates. 200 μL of sample solution was transferred into the 96-well UV plate (Costar® 3370, flat bottom, sterile polystyrene, Corning Lifesciences, Corning, NY, USA).

3. Synthesis of arginine-reactive mPEGs

3.1. Synthesis of mPEG tosylate

mPEG tosylate with molecular weights of 0.35 kDa, 1 kDa, and 2 kDa were all prepared using the same protocol. As a representative example, mPEG (4 g, 11.42 mmol) was dissolved in 120 mL of toluene and 60 mL of dichloromethane. *p*-Toluenesulfonyl chloride (11.44 g, 60 mmol) was added thereto, followed by the addition of 7.5 mL of triethylamine. The mixture was stirred for 6 h at r.t., and was followed by a second addition of *p*-toluenesulfonyl chloride (11.44 g, 60 mmol). The reaction was pursued for another 10 h, insoluble matter was filtered off, and the filtrate was concentrated under reduced pressure. The residue was purified by silica gel chromatography using mixtures of dichloromethane and methanol as eluent, yielding 4.04 g of the desired product (8.08 mmol, 70.7%). The recovered yields were 93% and 62% when using 1 kDa and 2 kDa, respectively. Fully assigned ^{1}H and ^{13}C NMR spectra can be found Figures S1–S3.

3.2. Synthesis of 4'-mPEG-2'-substituted-acetophenone (**1a–d**)

Compounds **1a–d** were all prepared using the same protocol. As a representative example, mPEG tosylate (1 g, 2 mmol) and 4-hydroxyacetophenone (2.72 g, 20 mmol) were dissolved in 100 mL of *N*,*N*-dimethylformamide. Then, potassium carbonate (2.76 g, 20 mmol) was added and the mixture was stirred for 4 h at 120 °C. Following this, the insoluble matter was filtered off with filter paper and the filtrate concentrated under reduced pressure. The residue was purified by silica gel chromatography using mixtures of dichloromethane and methanol as eluent, and the desired product dried *in vacuo*. The recovered yields were 75%, 74%, and 81% for **1a** (0.35 kDa, 1 kDa and 2 kDa, respectively) and 61%, 51%, and 65% for **1b–d** (0.35 kDa), respectively. Fully assigned ^{1}H and ^{13}C NMR spectra of **1a–d** can be found Figures S4–S9.

3.3. Synthesis of 4'-mPEG-2'-substituted-phenylglyoxal (**2a–d**)

Compounds **2a–d** were all prepared using the same protocol. As a representative example, **1a** (0.50 g, 1 mmol) was dissolved in 30 mL 1,4-dioxane, and selenium dioxide (0.33 g, 3 mmol) was then carefully added to the solution at room temperature. Then solution was heated to reflux for 4 h, after which time it was cooled, the insoluble matter removed by filtration (paper) and the filtrate concentrated under reduced pressure. Compounds **2a–d** were purified from the resulting mixture by preparative high-performance liquid chromatography and recovered by lyophilisation. The recovered yields were 41%, 92%, and 87% for **2a** (0.35 kDa, 1 kDa and 2 kDa, respectively) and 61%, 56%, and 66% for **2b–d** (0.35 kDa), respectively. Fully assigned ^{1}H NMR, ^{13}C NMR, and MS spectra of **2a–d** can be found Figures S10–15.

3.4. PEGylation of **3–5** and control peptides with **2a–d**

The kinetics of PEGylation of **3–5** and control peptides with **2a–d** were analyzed using the same protocol. As a representative example, 0.1 mg of **3** (1 eq.) was dissolved in 0.8 mL of 100 mM phosphate buffer pH 7.4. **2a** was dissolved into 100 mM sodium phosphate buffer pH 7.4 (or 100 mM borate pH 9) to prepare a 0.83 M stock solution. Then, 0.2 mL **2a** stock solution (4 eq. relative to arginine) was added using a micropipette to the peptide solution, which was then gently agitated and left to incubate for 24 h at r.t. Note: Control peptides do not contain arginine, but were considered to have one arginine residue for the purposes of calculating equivalents. During this period, 10 μL aliquots were withdrawn to monitor the progress of the

reaction by HPLC using the fluorescence detector. Kinetics were monitored in triplicate. The resulting conjugates were purified after the 24 h period by preparative HPLC, lyophilized, and stored at – 20 ºC. Representative chromatograms of the PEGylation process, kinetics, and the annotated MS of the formed conjugates can be found in Figures S16–25, 27, and 28.

3.5. PEGylation of short peptides with **2a–d**

The kinetics of PEGylation of the short arginine-containing peptides with **2a–d** was analyzed using the same protocol. As a representative example, 0.63 mg of peptide GGRGGW (1 µmol, 1 eq.) was dissolved in 1 mL 100 mM phosphate buffer pH 7.4, then **2a** (1.5 µmol, 1.5 eq., 1.80 µL of a 0.83 M **2a** stock solution) was added into the solution and then incubated at r.t. for 120 h. Small aliquots 10 µL were withdrawn to monitor the reaction by HPLC.

3.6. De-PEGylation

0.1 mg of purified conjugates were dissolved in 0.5 mL of 100 mM phosphate buffer pH 7.4 containing 10% fetal bovine serum (FBS). De-PEGylation under acidic and basic conditions were performed by adjusting the pH of the phosphate buffer to 4 and 9 using hydrochloric acid or sodium hydroxide, respectively. The de-PEGylation process was monitored over 24 h by taking small aliquots (10 µL) and quantifying the amount of peptide release by HPLC using the fluorescence detector. Note that to avoid artefacts for bleaching of the fluorophore, the intensity of the released peptide peak was normalized to the total intensity of the entire chromatogram. De-PEGylation in 50% serum was performed by extension of the protocol above, with the addition of a protein-removal step prior to analysis by HPLC. In brief, 40 µL of the reaction mixture solution was added 100 µL acetonitrile containing 0.1 % trifluoroacetic acid and mixed gently. The mixture was kept at room temperature for 10 min then centrifuged at 6,800 × g for 10 min. The supernatant was removed and transferred into HPLC vials for analysis. Kinetics were all performed in triplicate.

4. Determination of the degree of PEGylation for [3+2a–c] conjugates

Determining the average degree of PEGylation of the conjugates [**3+2a–c**] requires quantification of the number of moles of the peptide component as well as the number of moles of the mPEG component, for a given dry weight of the conjugate. To determine peptide content, a known mass of conjugate was dissolved in 500 µL D_2O, and the absorbance of the solution at 452 nm (the FITC dye on the peptide) was compared to a standard curve obtained with the pure peptide. Then, to determine the number of moles of mPEG within this solution, a small amount (5 µL) of dimethyl sulfoxide was quantitatively added to a known volume of the D_2O solution, and a ^1H NMR spectrum recorded. Comparing the integration of the peaks of the mPEG main-chain (~3.6 ppm) relative to those of DMSO provided access to the number of moles of mPEG within the solution. The degree of PEGylation is calculated by dividing the number of moles of mPEG by the number of moles of peptide. The number of mPEG chains per molecule of **3** was 1.5, 1.3, and 2.0 for **2a**, **2b**, and **2c**, respectively.

5. Antibacterial assay

The antibacterial activity of AMP **3** and its derivatives was determined using the broth micro dilution method. Briefly, a single colony of *E. coli B* (*Escherichia coli* B, HER1024, obtained from the Felix d'Herelle Reference Center for Bacterial Viruses, Université Laval, Quebec) was inoculated into 10 mL of Luria-Bertani broth (LB broth) and cultured overnight at 37 ºC. The

overnight culture was diluted 1000-fold with fresh LB broth and incubated at 37 ºC for 4.5 h to obtain mid-logarithmic phase organisms (10^9 cfu·mL^{-1}). This solution was diluted to 10^7 cfu·mL^{-1} with LB broth, and 90 µL aliquots were pipetted into each well of a 96-well plate. 10 µL of analyte solution were then added to each well. Ten-µL of PBS alone or ampicillin (1mg·mL^{-1} in PBS) served as negative and positive controls, respectively. Samples for analysis were prepared by reacting AMP **3** (5 mM) with either 0 (control) or 12 molar eq. of **2a** or **2c** (2 kDa) in 120 µL of PBS, pH 7.4, at room temperature for 48 h. This solution was diluted to 1 mL with full human serum (final solution contains 88% serum) containing 0.01 wt% trypsin (>9 kU·mg^{-1} for N_α-Benzoyl-L-arginine ethyl ester hydrochloride), and incubated at r.t. for 1 h, 6 h, or 24 h. After this period, proteins were immediately removed by addition of 1.5 mL acetonitrile, sedimentation of precipitate by centrifugation, quantitative recovery of the supernatant, and drying by freeze-drying (FreeZone 4.5, Labconco). The dried samples were reconstituted in 60 µL PBS to yield a stock solution containing 10 mM of **3** (total of intact and potentially degraded AMP **3**). This solution was serially diluted with PBS to 500 µM – 10 nM (final concentration in well). After addition of analyte to the well, the microplate was incubated at 37 ºC for 16 h with shaking, and growth inhibition was determined by measuring absorbance at 620 nm using a Cytation 5 multi-mode microplate reader (BioTek Inc., VT). IC_{50}s were determined by non-linear regression using the "dose-response" function in Microcal Origin, and are the mean of 3–4 independent experiments. Fresh AMP **3**, and [**3+2a**]/[**3+2c**] incubated in PBS for 6 h served as controls for the experiments above.

6. Cytotoxicity assay

HepG2 cells were grown in MEM with GlutaMAX™ supplemented with 10 vol% FBS and 1 vol% Penicillin-Streptomycin mixture. Stock cultures were maintained at 37 ºC in a humidified atmosphere containing 5 % CO_2 and passaged every 2–4 days using 0.25 % trypsin/EDTA solution. Cells were seeded 24 h prior the experiments in 96 well tissue culture plates (TPP, Trasadingen, CH) at a density of 15,000 cells per well (48,000 cells·cm^{-1}). Cytotoxicity experiments were carried out in MEM without L-glutamine and without Phenol Red supplemented with 10 vol% FBS, 1 vol% GlutaMAX™ and 1 vol% penicillin streptomycin. All test compounds were dissolved in this medium at a concentration of 10 mM and further diluted to the final experimental concentrations with medium. Prior to addition of sample solution, cells were grown for 24 h, and then washed once with 100 µL PBS. At this point, the sample solutions (100 µL) or the medium alone (blank) were added. As positive control for the MTS assay, 1 mM H_2O_2 was used. Cells were exposed to the test substances for 3 h at 37 ºC. The sample solutions were then removed, 120 µL of diluted MTS solution was then added (20 µL MTS solution from the kit were diluted with 100 µL medium) and incubated at 37 ºC for 2 h. Finally, the absorbance at 490 nm was measured using a plate reader (Infinite® M200, Tecan). For each compound, three independent experiments were performed, and each experiment was carried out in triplicates. MTS solution was used as absorbance background. From all absorbance values, the background of MTS incubated at 37 ºC for 2 h without any cells was subtracted. Cells only exposed to medium was chosen as 100 % viability, and all values were normalized to it.

5. Supporting results

Table S1. Multiple pairwise means comparison table for Figure 3 (One-way ANOVA, Tukey post hoc p = 0.05). The value "1" indicates that the difference of the means is significant at the 0.05 level. The value "0" indicates that the difference of the means is not significant at the 0.05 level.

**2a**		*Peptide*				
		KKRKK	GKRKG	GGRGG	GEREG	EEGEE
	KKRKK	0				
	GKRKG	0	0			
Peptide	GGRGG	1	1	0		
	GEREG	0	0	1	0	
	EEREE	0	0	1	0	0

**2b**		*Peptide*				
		KKRKK	GKRKG	GGRGG	GEREG	EEGEE
	KKRKK	0				
	GKRKG	1	0			
Peptide	GGRGG	1	1	0		
	GEREG	1	1	1	0	
	EEREE	0	1	0	1	0

**2c**		*Peptide*				
		KKRKK	GKRKG	GGRGG	GEREG	EEGEE
	KKRKK	0				
	GKRKG	0	0			
Peptide	GGRGG	1	1	0		
	GEREG	0	1	1	0	
	EEREE	0	0	1	0	0

**2d**		*Peptide*			
		KKRKK	GKRKG	GGRGG	GEREG
	KKRKK	0			
	GKRKG	0	0		
Peptide	GGRGG	1	1	0	
	GEREG	1	1	1	0

Table S2. Non-linear regression of de-PEGylation data from Figure 4a. Parameters "*A*" represent the magnitude of release, while parameters "*t*" represent the width of the release. Subscript "*1*" corresponds to the burst release phase of the release, while subscript "*2*" corresponds for release from form **7** of the conjugates (with the exception of **2a** for which release from form **7** and **8** cannot be distinguished). Parameters (and error) in Table S2 were obtained by non-linear regression performed on the average kinetics (Mean ± SD, n = 3).

$$y = y_0 + A_1(1 - e^{-x/t_1}) + A_2(1 - e^{-x/t_2})$$

	y_0	A_1 (%)	t_1 (h)	A_2 (%)	t_2 (h)
**2a**	0 ± 0.05	19 ± 1	0.06 ± 0.02	70 ± 1	9 ± 1
**2b**	0 ± 0.02	33 ± 2	0.09 ± 0.01	28 ± 2	1.9 ± 0.3
**2c**	0 ± 0.007	17.1 ± 0.8	0.062 ± 0.08	27 ± 2	11 ± 2
**2d**	0 ± 0.02	34.2 ± 0.4	0.03 ± 0.06	2e14 ±6e15	3e14 ± 8e15

*:

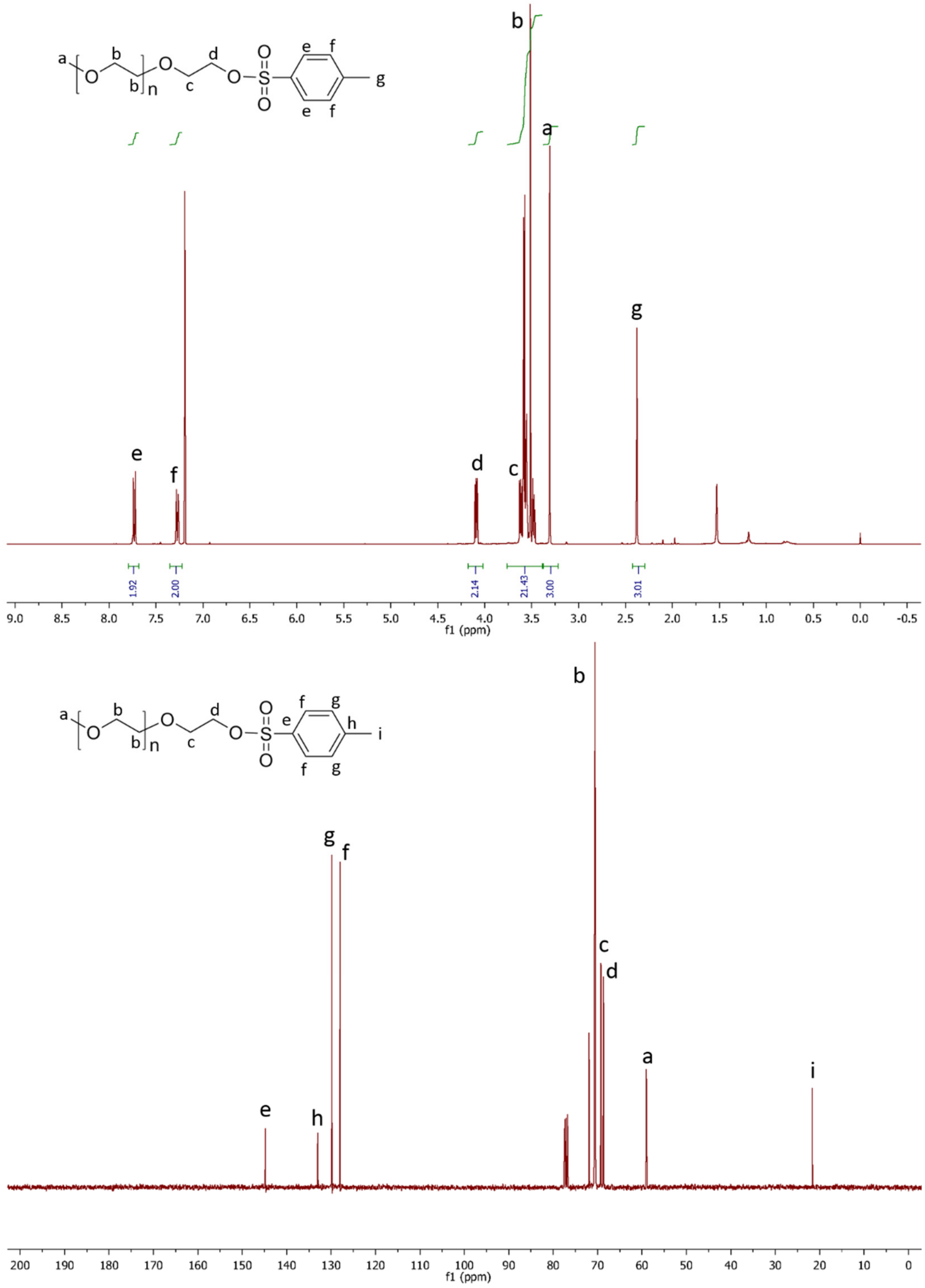

Figure S1. Assigned ^{1}H and ^{13}C NMR spectra of mPEG tosylate (0.35 kDa) in $CDCl_3$.

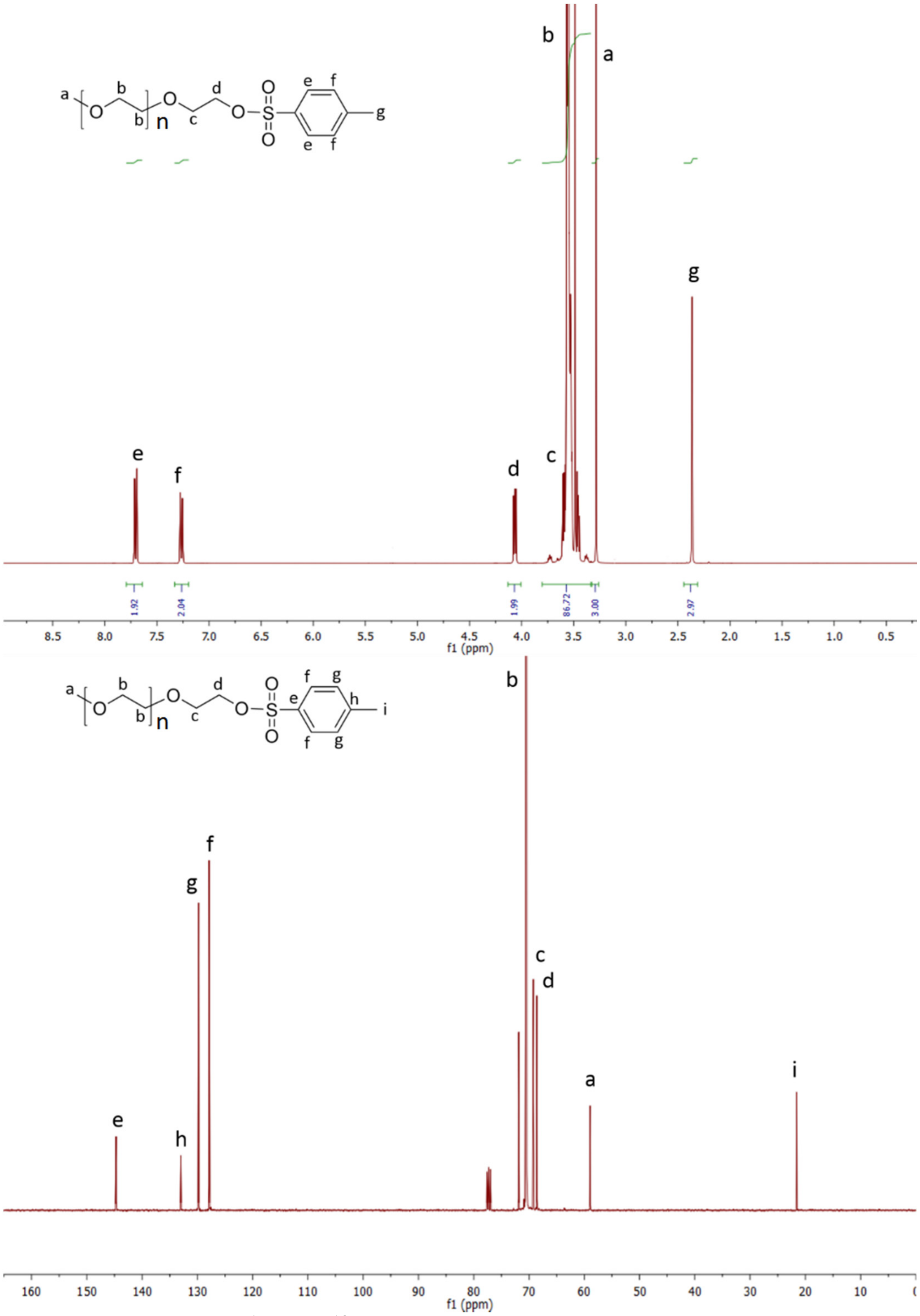

Figure S2. Assigned ^{1}H and ^{13}C NMR spectra of mPEG tosylate (1 kDa) in D_2O.

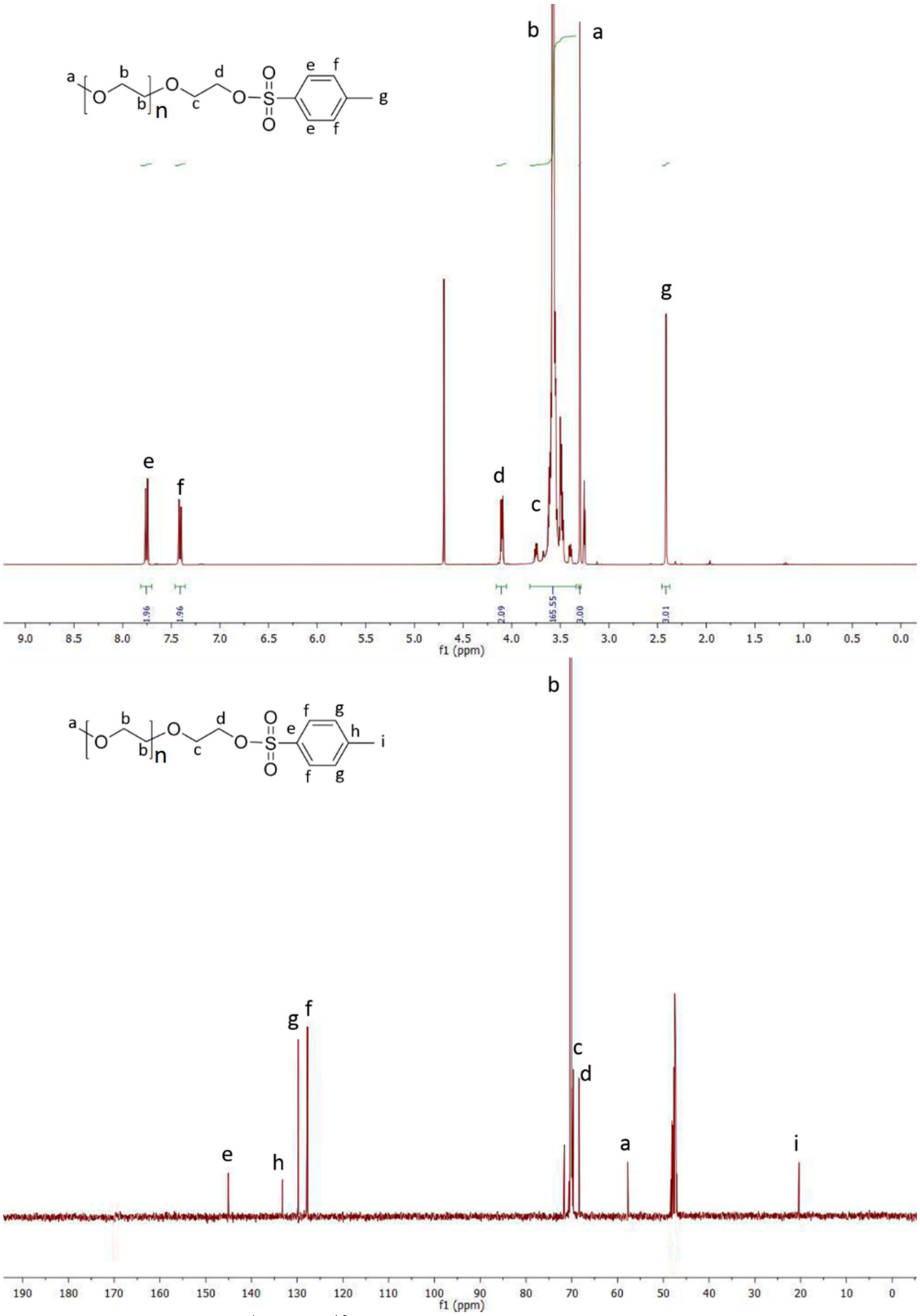

Figure S3. Assigned ^{1}H and ^{13}C NMR spectra of mPEG tosylate (2 kDa) in D_2O.

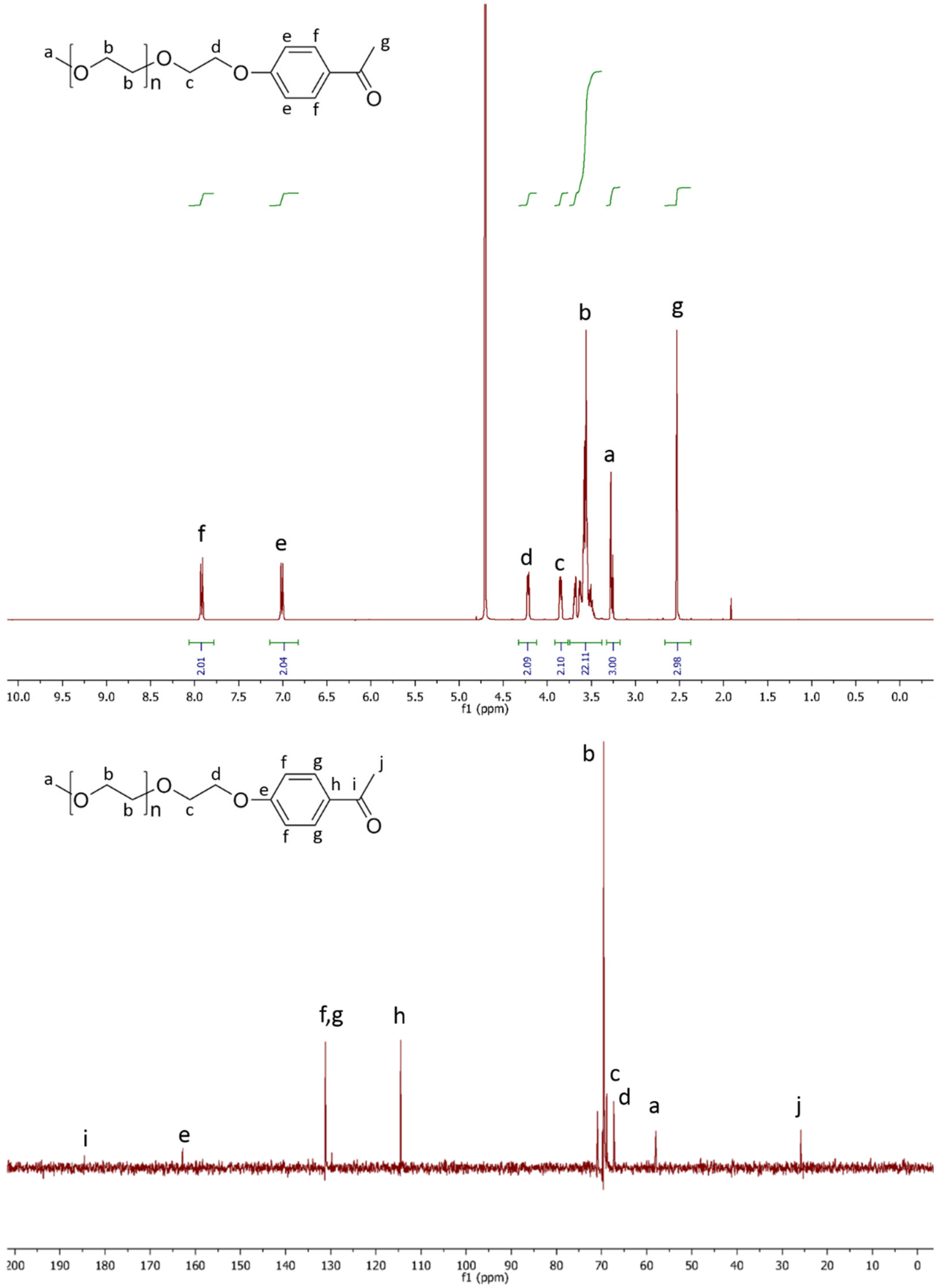

Figure S4. Assigned ^{1}H and ^{13}C NMR spectra of **1a** (0.35 kDa) in D_2O.

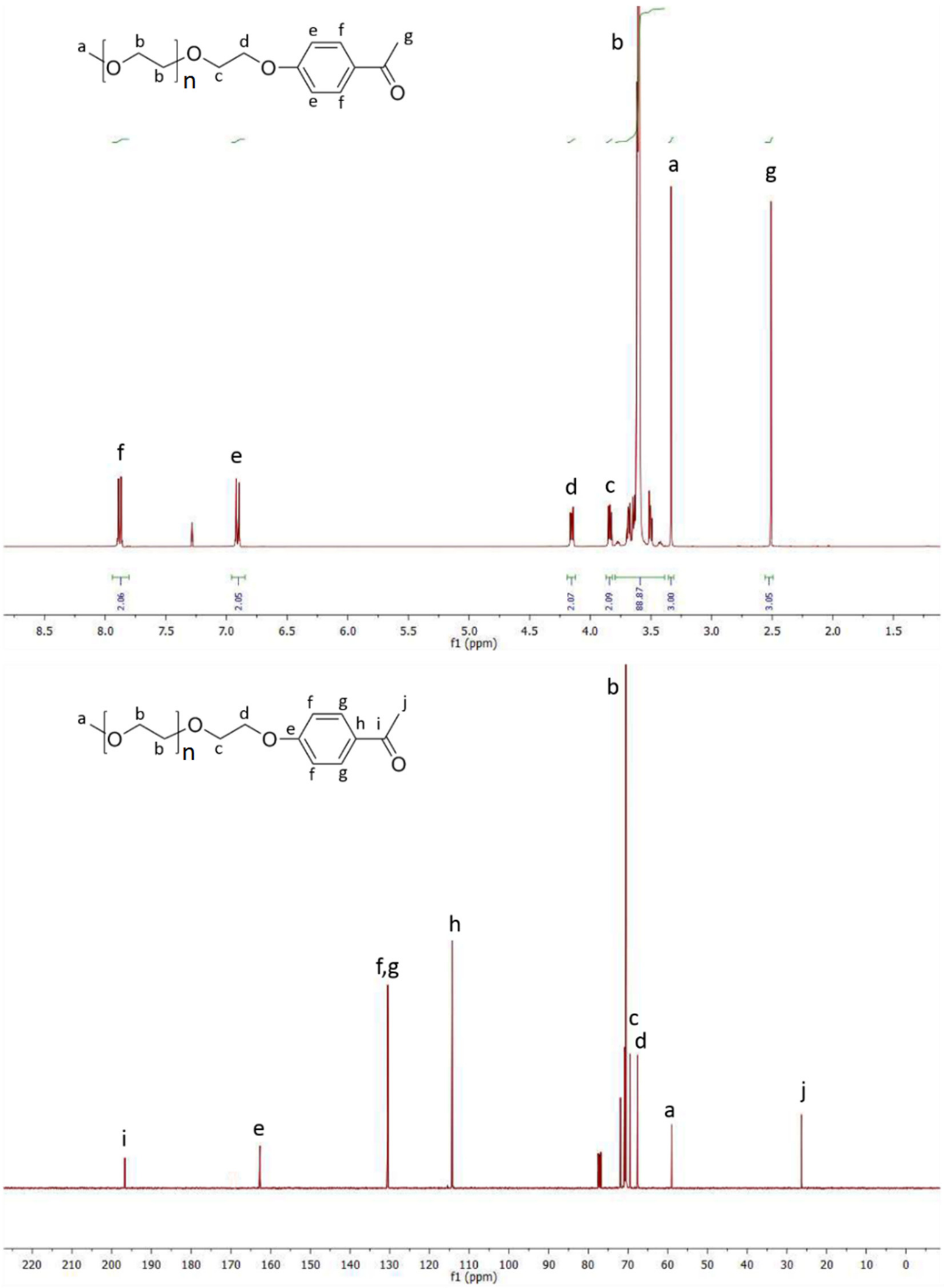

Figure S5. Assigned ^{1}H and ^{13}C NMR spectra of **1a** (1 kDa) in $CDCl_3$.

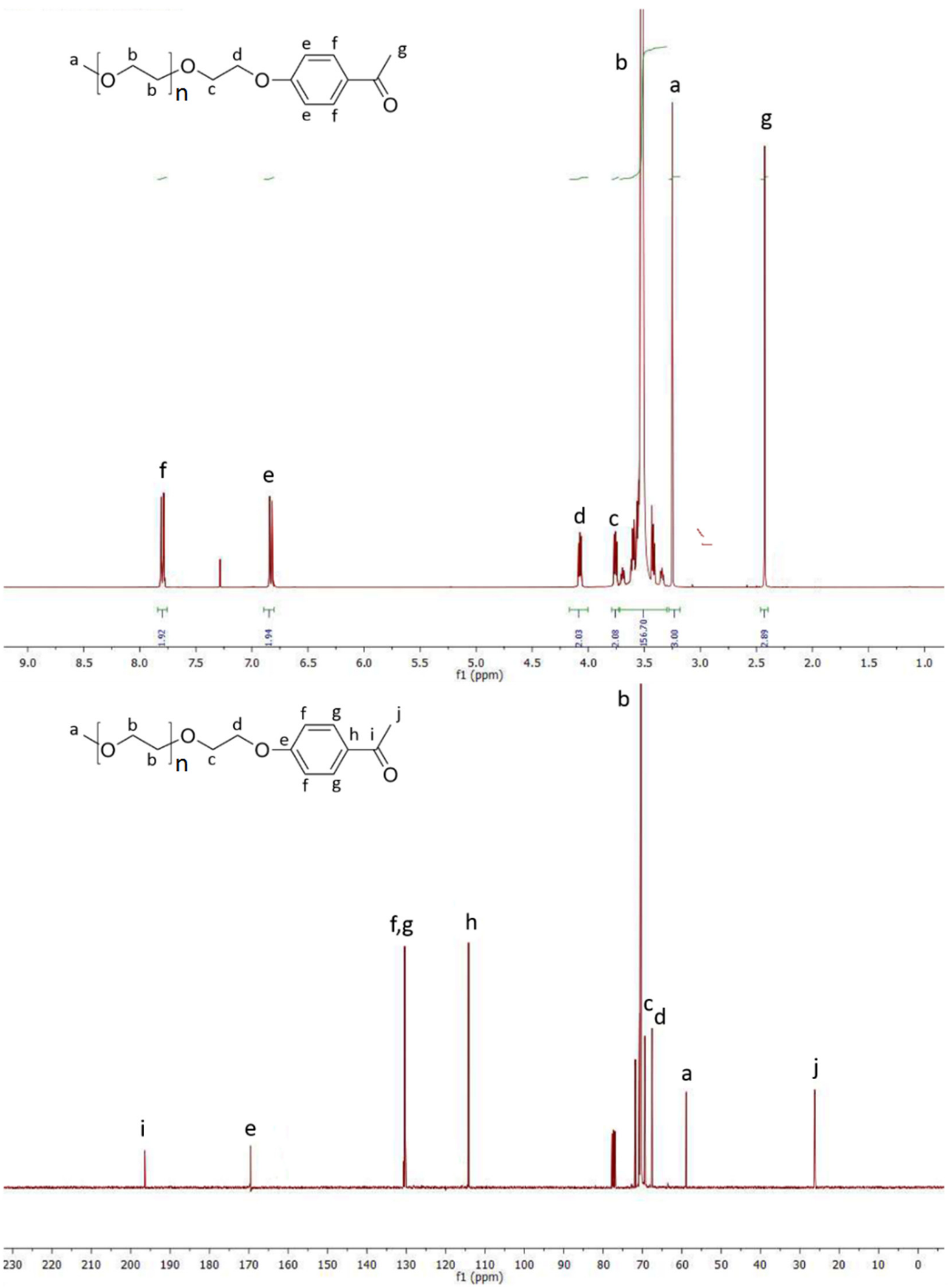

Figure S6. Assigned ^{1}H and ^{13}C NMR spectra of **1a** (2 kDa) in $CDCl_3$.

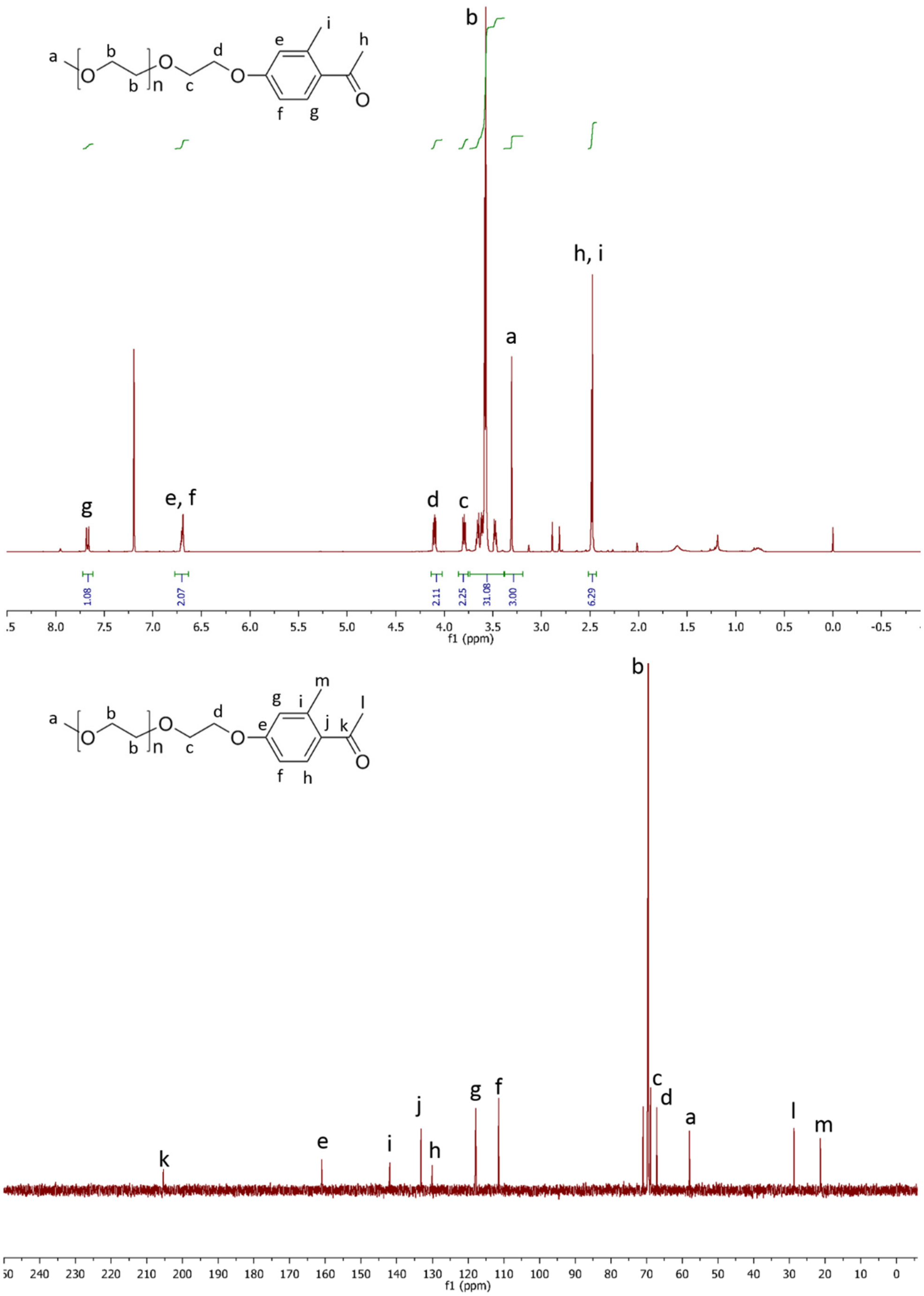

Figure S7. Assigned ^{1}H and ^{13}C NMR spectra of **1b** (0.35 kDa) in $CDCl_3$.

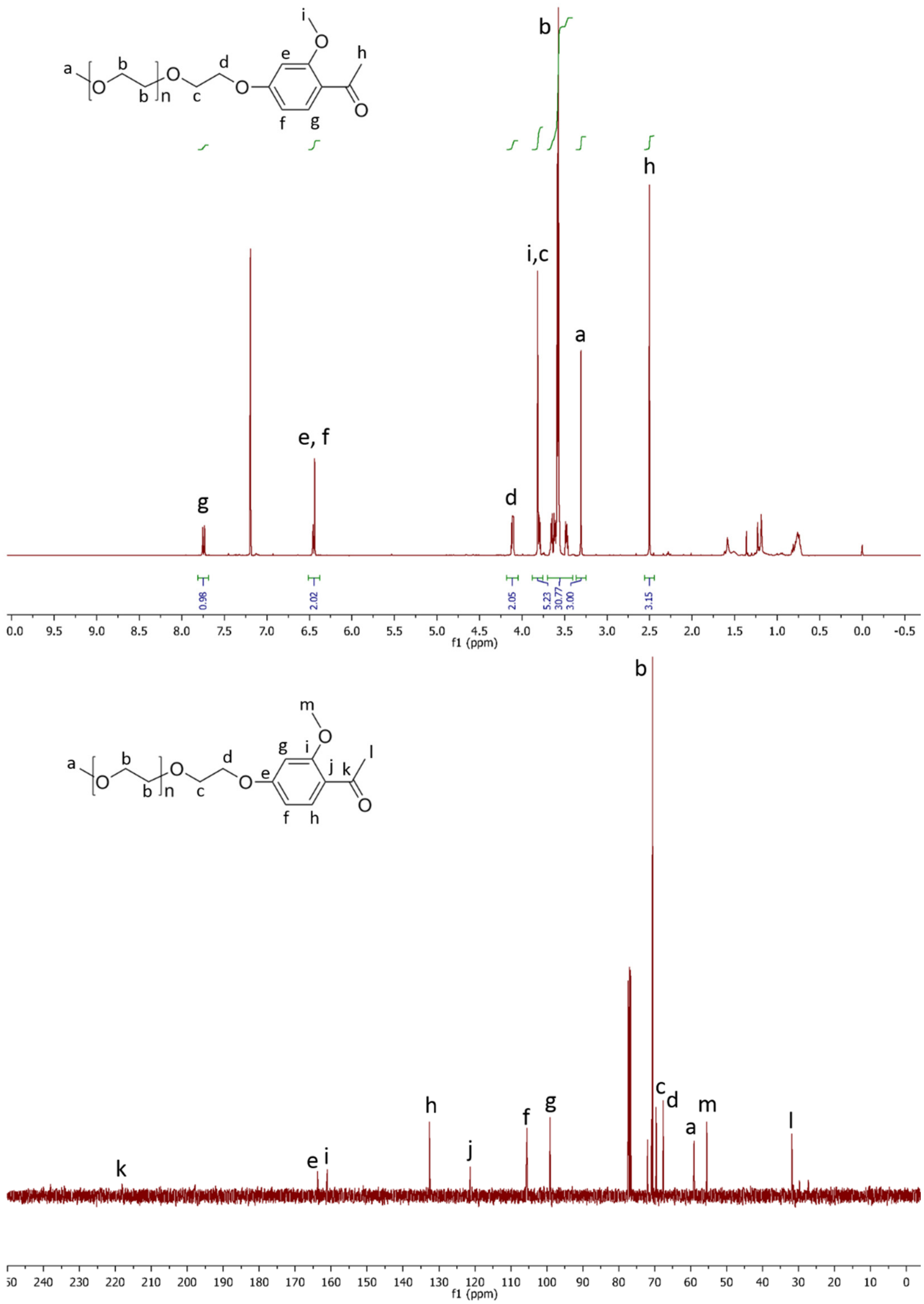

Figure S8. Assigned ^{1}H and ^{13}C NMR spectra of **1c** (0.35 kDa) in $CDCl_3$

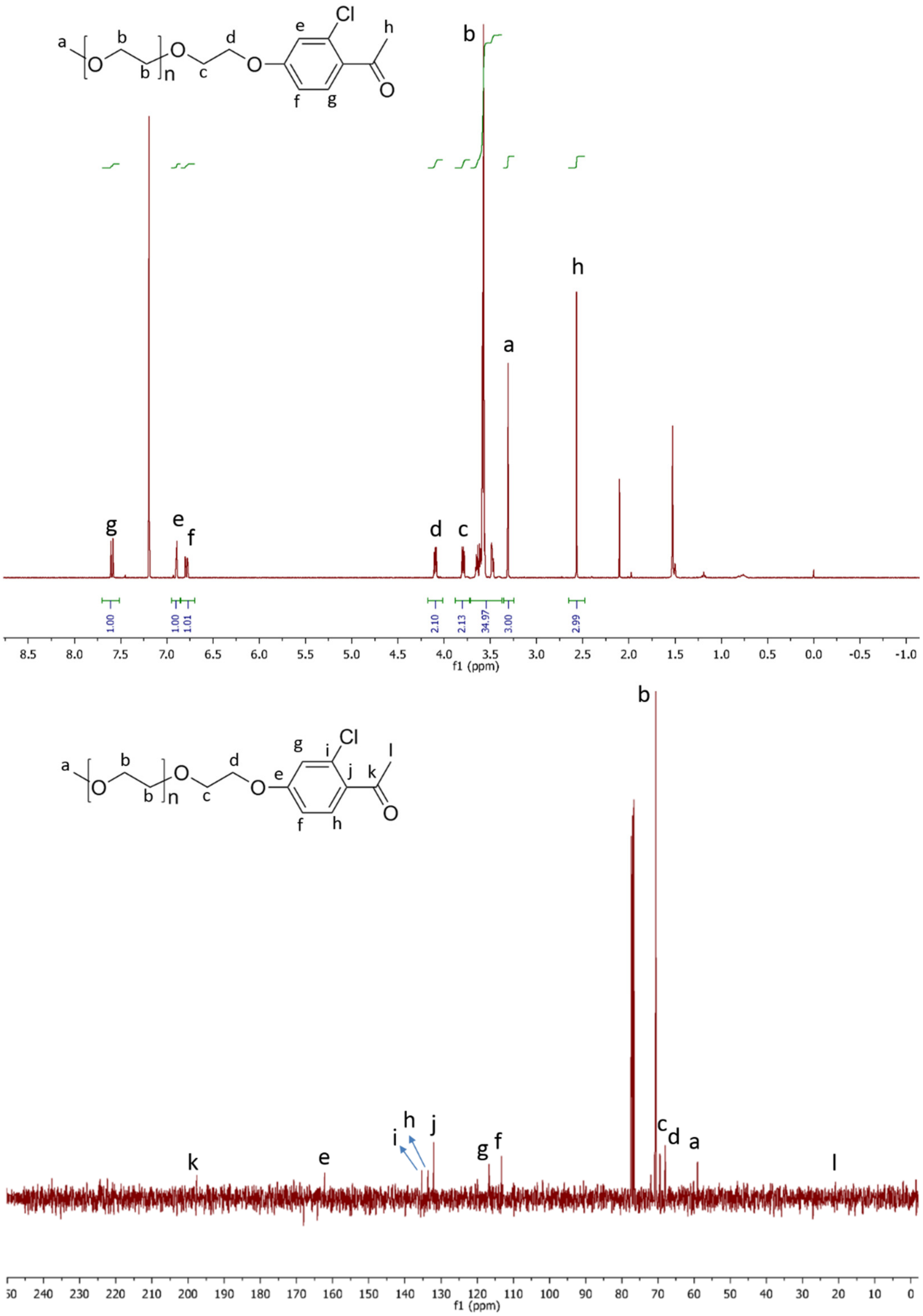

Figure S9. Assigned ^{1}H and ^{13}C NMR spectra of **1d** (0.35 kDa) in $CDCl_3$.

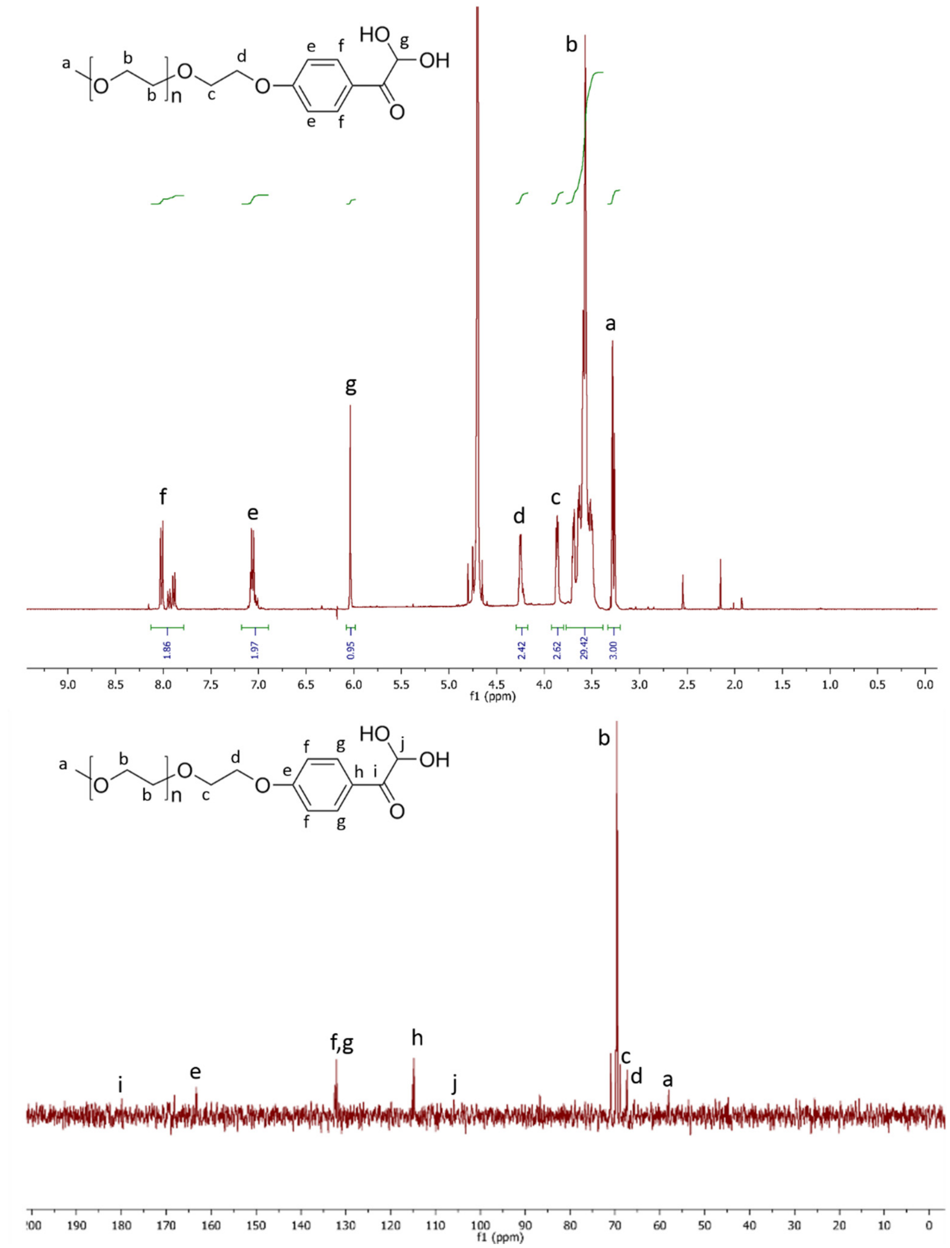

Figure S10a. Assigned ^{1}H and ^{13}C NMR spectra of **2a** (0.35 kDa) in D_2O.

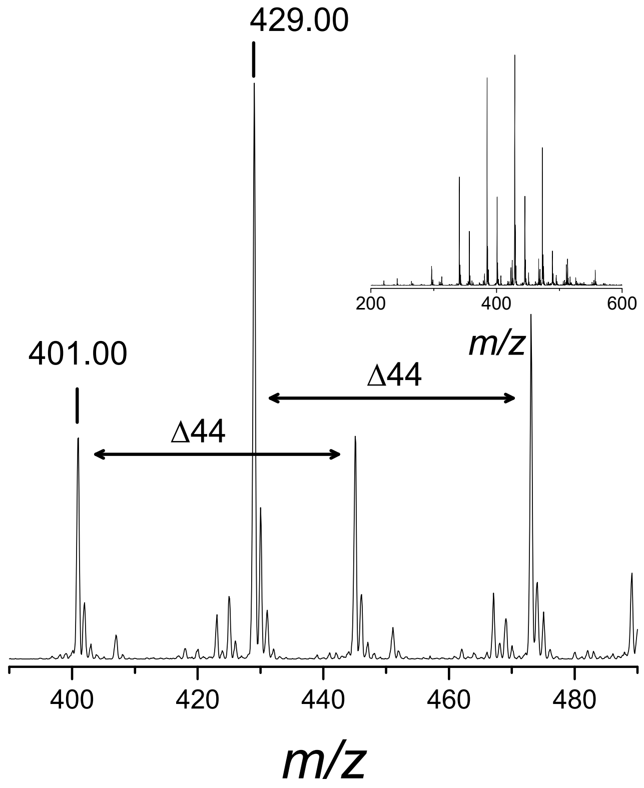

Figure S10b. Mass spectrum of **2a** (0.35 kDa). HRMS (ESI-ion trap) m/z: Parent ions: $[M + H]^+$ Calcd for $C_{21}H_{33}O_9$ 429.21 (Found 429.00). Fragment ions: [M + H]+ (α-cleavage at aldehyde) Calcd for $C_{20}H_{33}O_8$ 401.22 (Found 401.00).

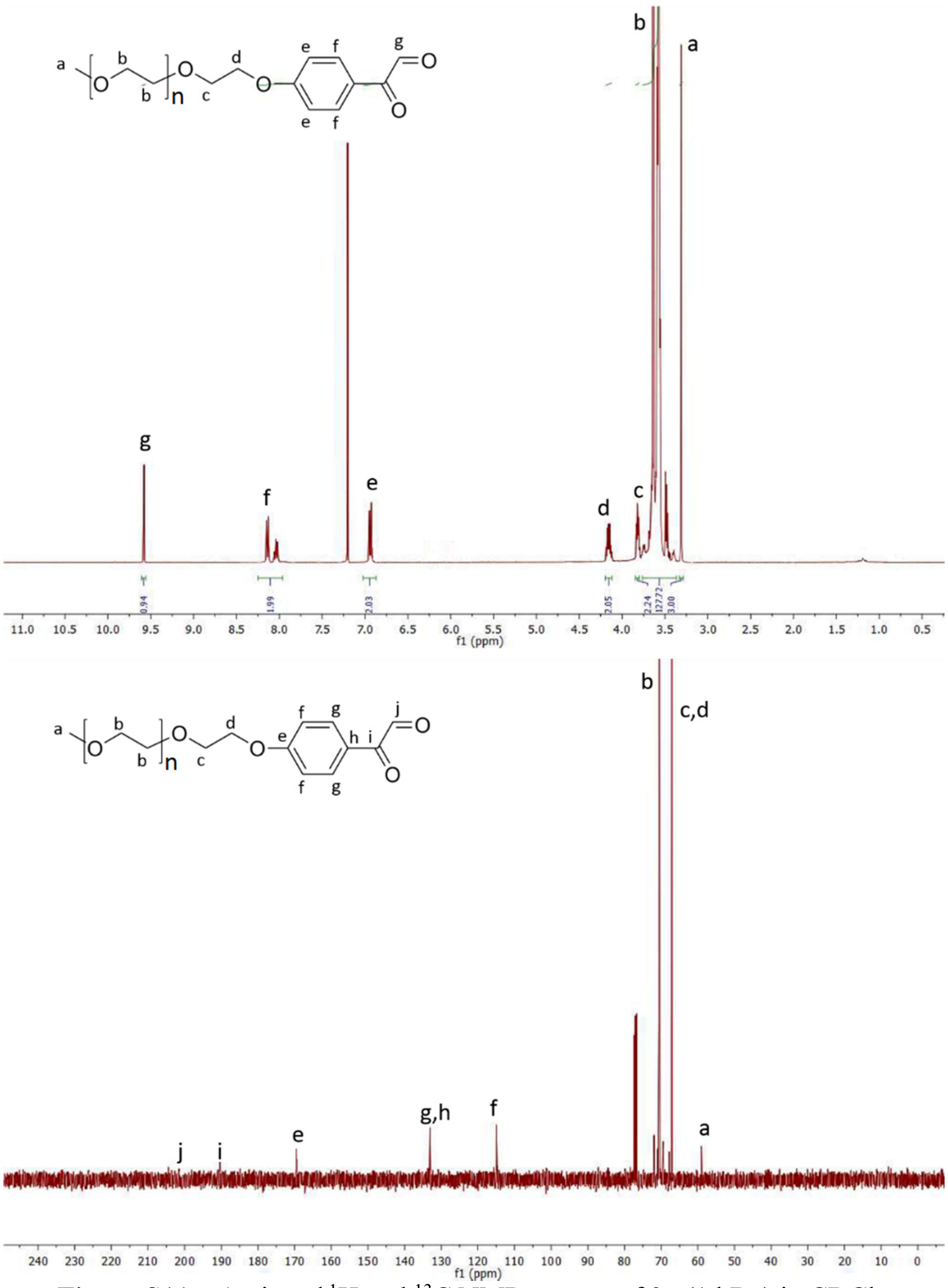

Figure S11a. Assigned ^{1}H and ^{13}C NMR spectra of **2a** (1 kDa) in $CDCl_3$.

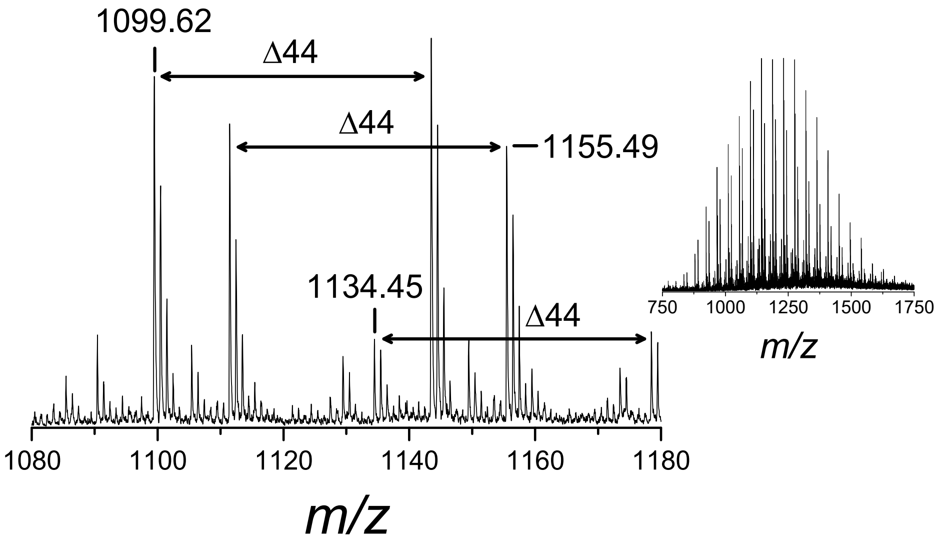

Figure S11b. Mass spectrum of **2a** (1 kDa). HRMS (MALDI-TOF) m/z: Parent ions: $[M + H]^+$ Calcd for $C_{53}H_{97}O_{25}$ 1133.63 (Found 1134.45); $[M + Na]^+$ Calcd for $C_{53}H_{96}NaO_{25}$ 1155.61 (Found 1155.49). Fragment ions: $[M + Na]^+$ (α-cleavage at ketone) Calcd for $C_{51}H_{96}NaO_{23}$ 1099.62 (1099.62).

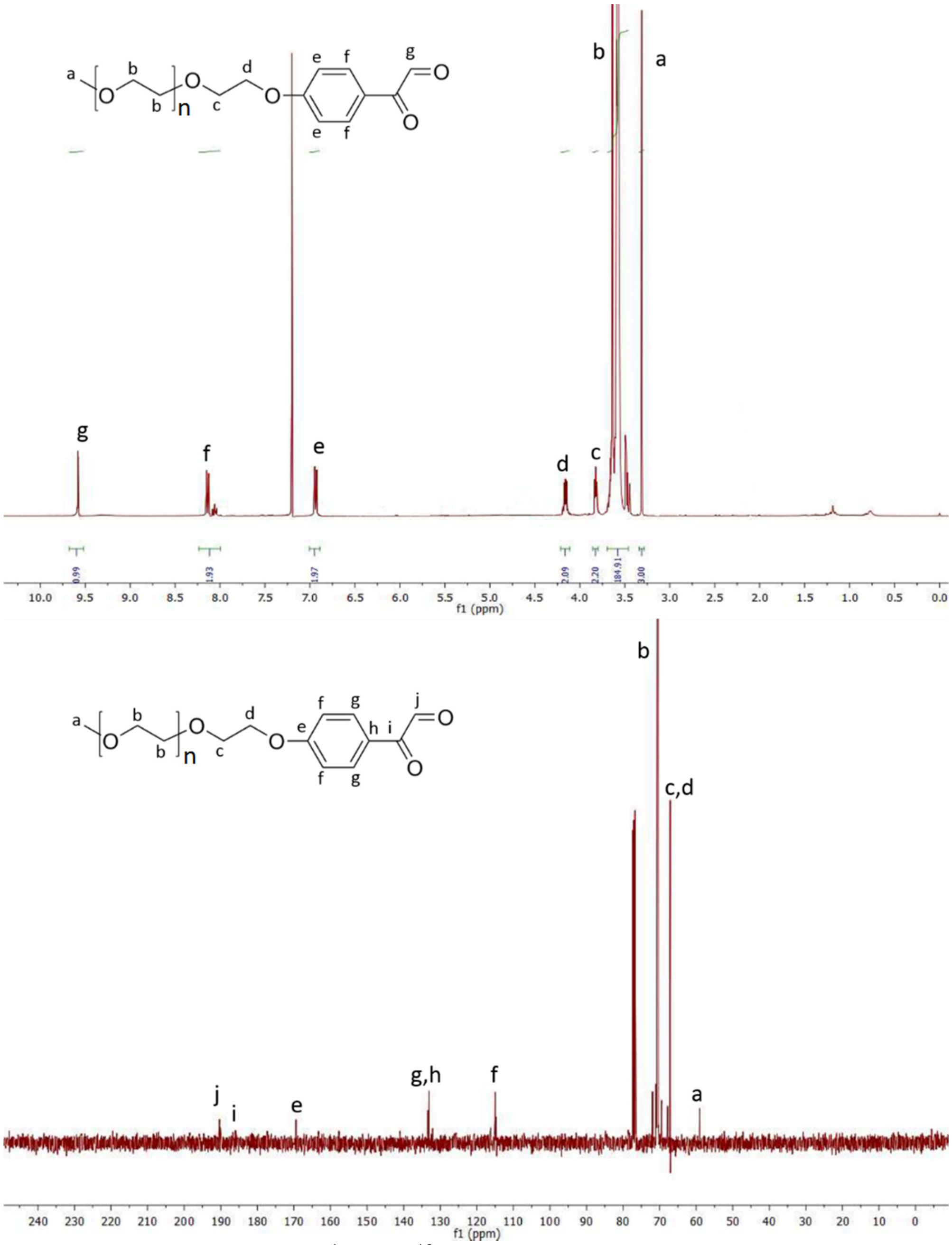

Figure S12a. Assigned ^{1}H and ^{13}C NMR spectra of **2a** (2 kDa) in $CDCl_3$.

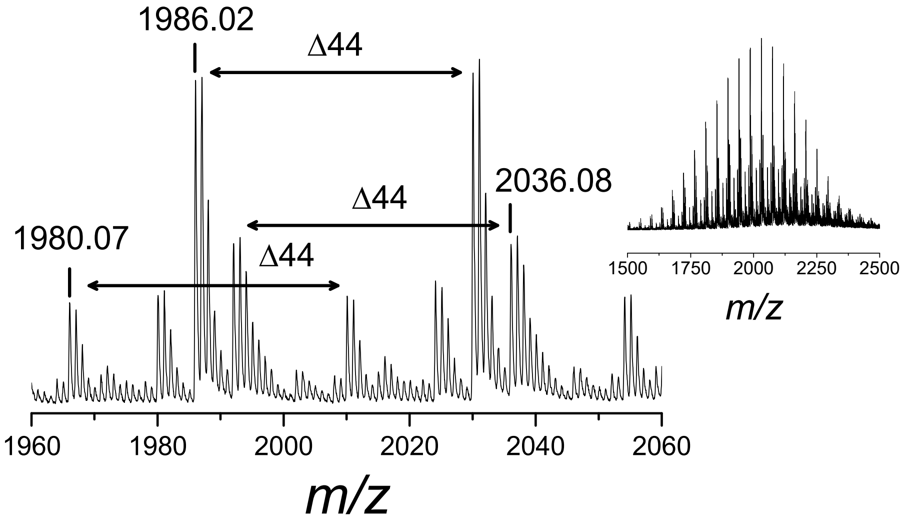

Figure S12b. Mass spectrum of **2a** (2 kDa). HRMS (MALDI-TOF) m/z: Parent ions: $[M + Na]^+$ Calcd for $C_{93}H_{176}NaO_{45}$ 2036.14 (Found 2036.08). Fragment ions: $[M + H]^+$ (α-cleavage at aldehyde) Calcd for $C_{92}H_{177}O_{44}$ 1986.16 (Found 1986.02); $[M + Na]^+$ (α-cleavage at ketone) Calcd for $C_{91}H_{176}NaO_{43}$ 1980.15 (Found 1980.07).

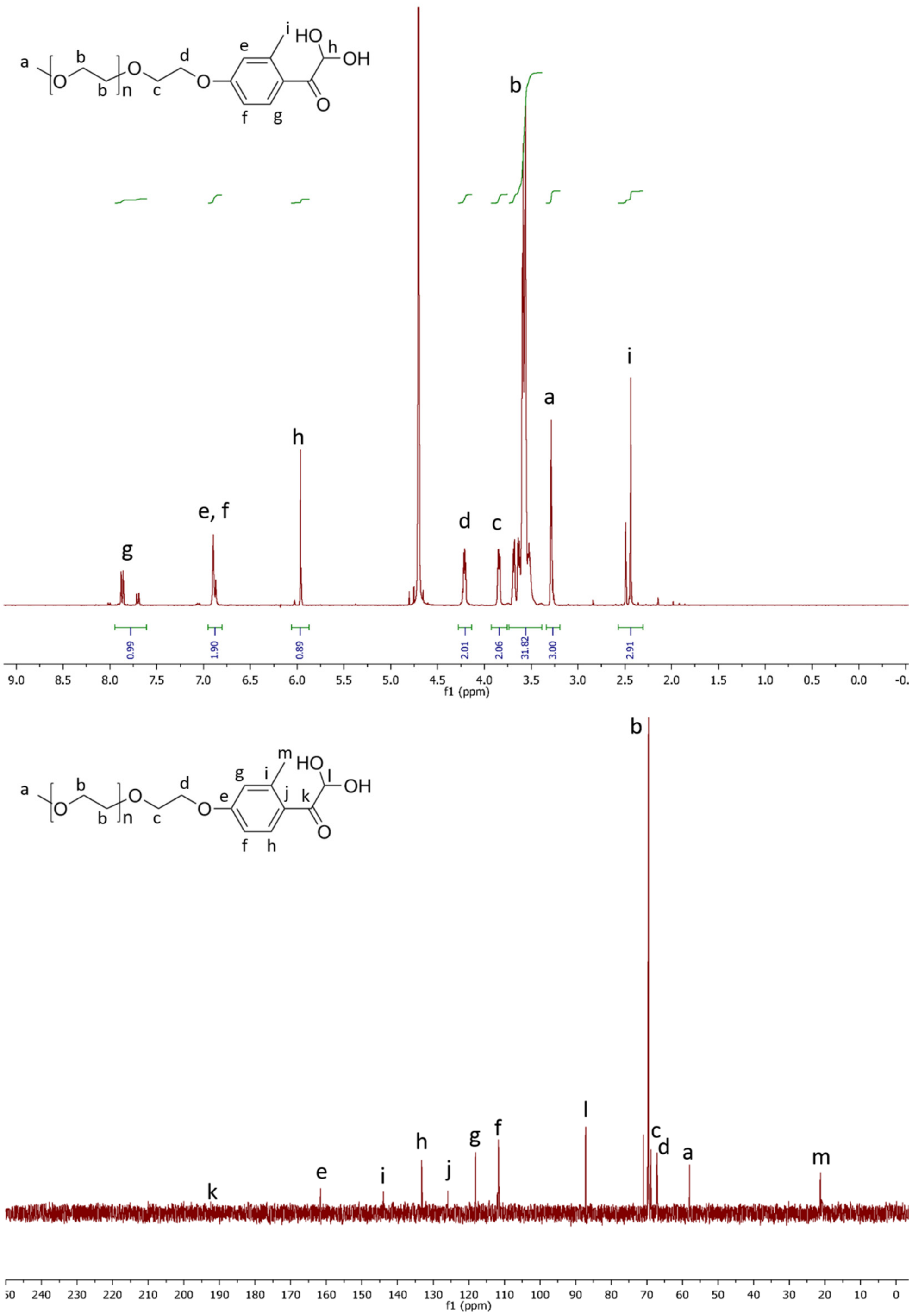

Figure S13a. Assigned ^{1}H and ^{13}C NMR spectra of **2b** (0.35 kDa) in D_2O.

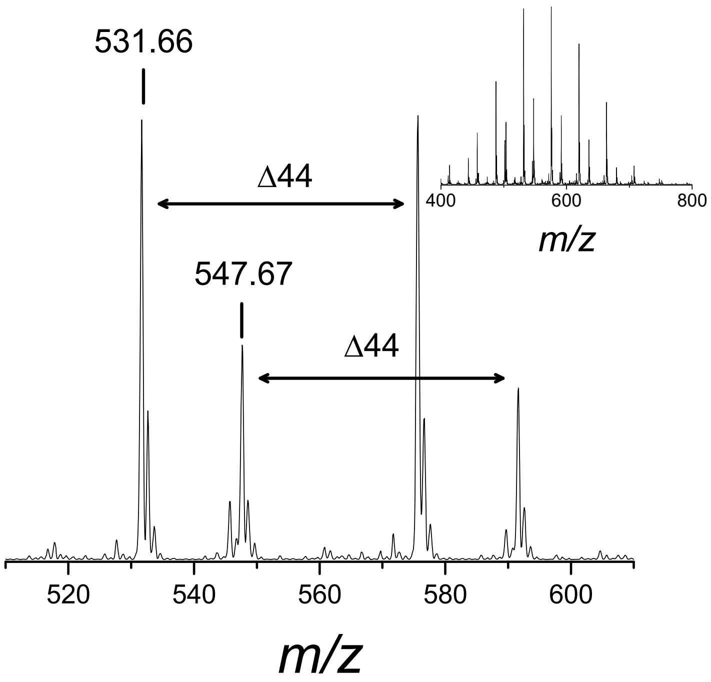

Figure S13b. Mass spectrum of **2b** (0.35 kDa). HRMS (ESI-ion trap) m/z: Parent ions: $[M + H]^+$ Calcd for $C_{26}H_{43}O_{11}$ 531.28 (Found 531.66). Fragment ions: [M + H]+ (α-cleavage at aldehyde) Calcd for $C_{27}H_{47}O_{11}$ 547.31 (Found 547.67).

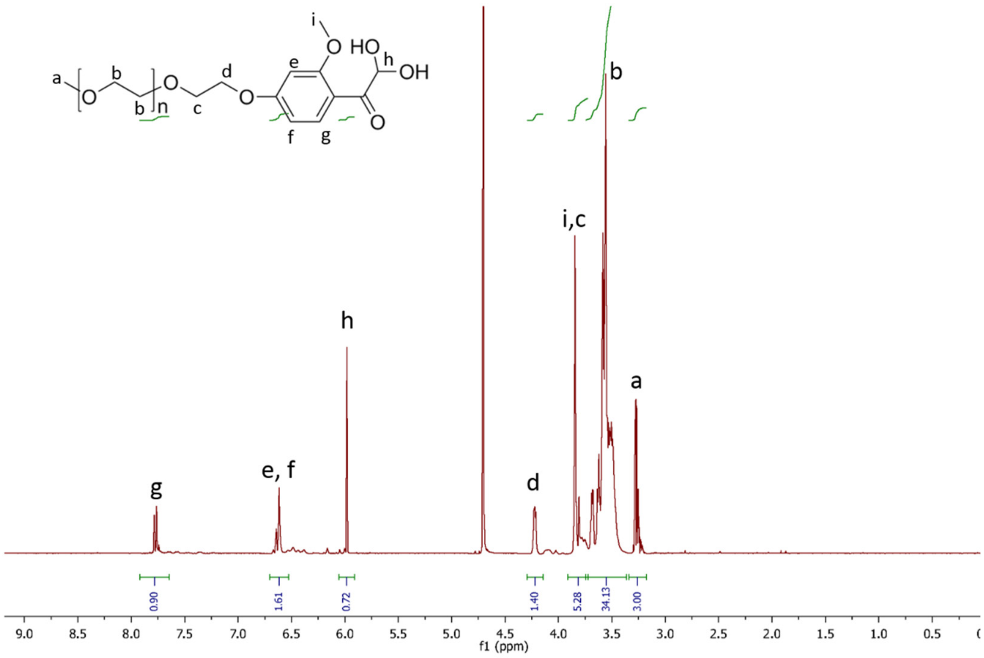

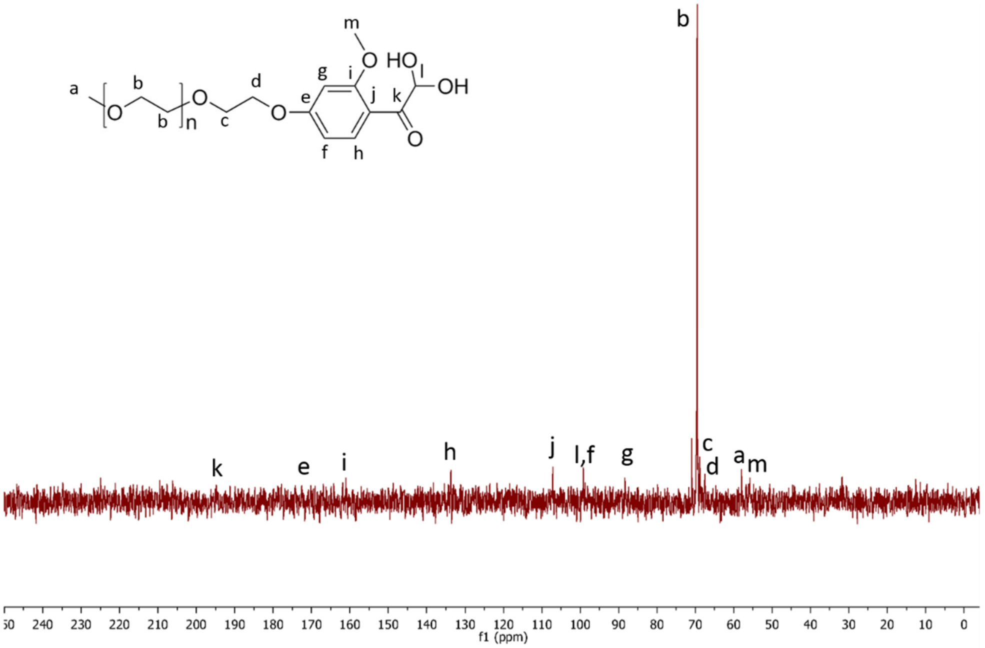

Figure S14a. Assigned ^{1}H and ^{13}C NMR spectra of **2c** (0.35 kDa) in D_2O.

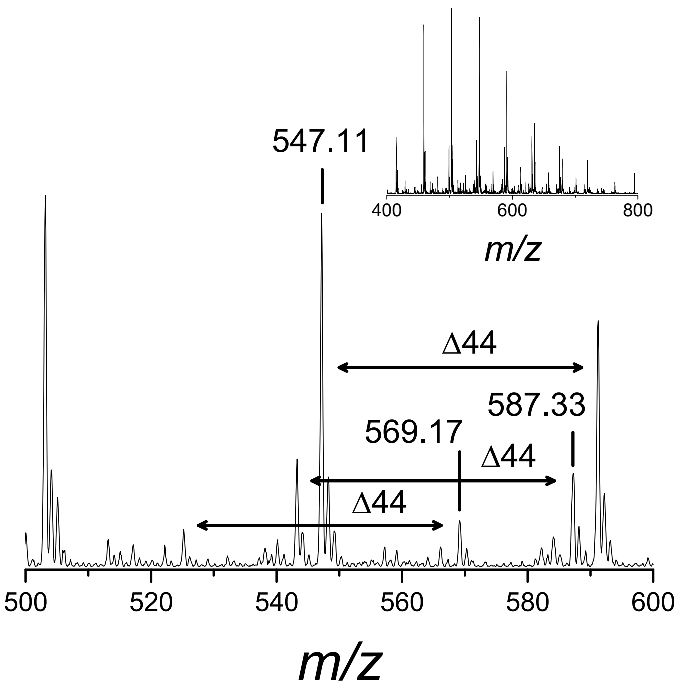

Figure S14b. Mass spectrum of **2c** (0.35 kDa). HRMS (ESI-ion trap) m/z: Parent ions: $[M + H]^+$ Calcd for $C_{26}H_{43}O_{12}$ 547.27 (Found 547.11); $[M + Na]^+$ Calcd for $C_{26}H_{42}O_{12}Na$ 569.26 (Found 569.17); $[M + H_2O + Na]^+$ Calcd for $C_{26}H_{44}O_{13}Na$ 587.27 (Found 587.33).

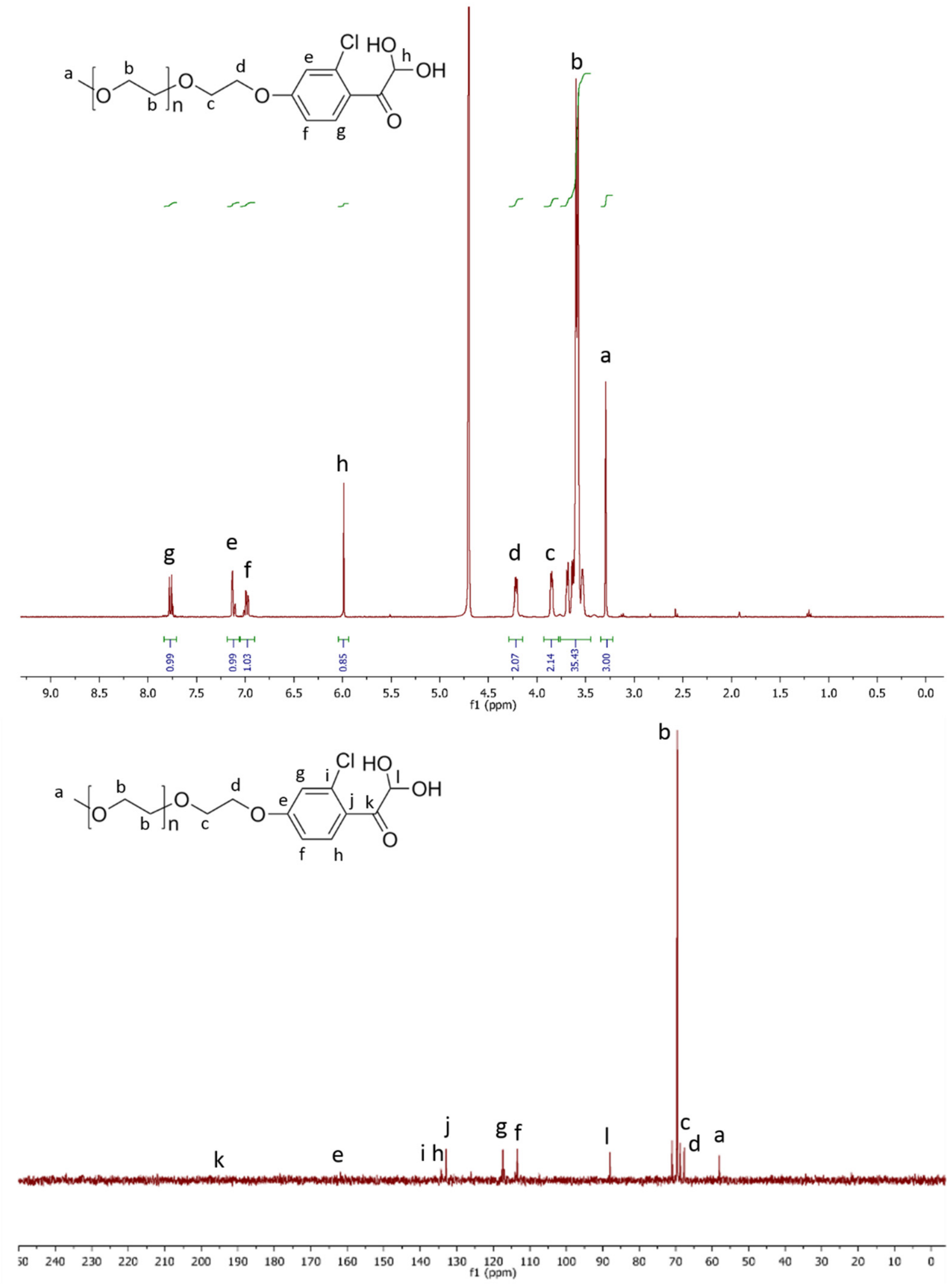

Figure S15a. Assigned ^{1}H and ^{13}C NMR spectra of **2d** (0.35 kDa) in D_2O.

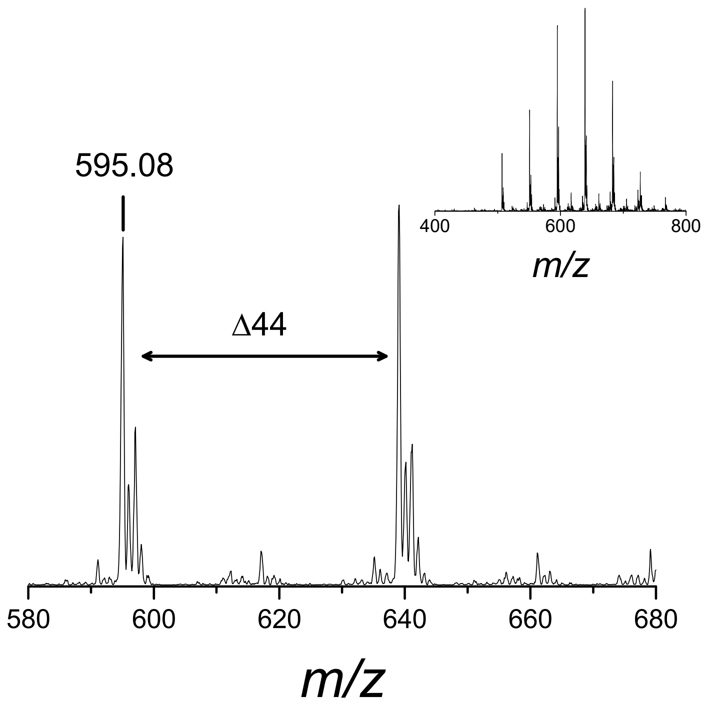

Figure S15b. Mass spectrum of **2d** (0.35 kDa). HRMS (ESI-ion trap) m/z: Parent ions: $[M + H]^+$ Calcd for $C_{27}H_{44}ClO_{12}$ 595.25 (Found 595.08).

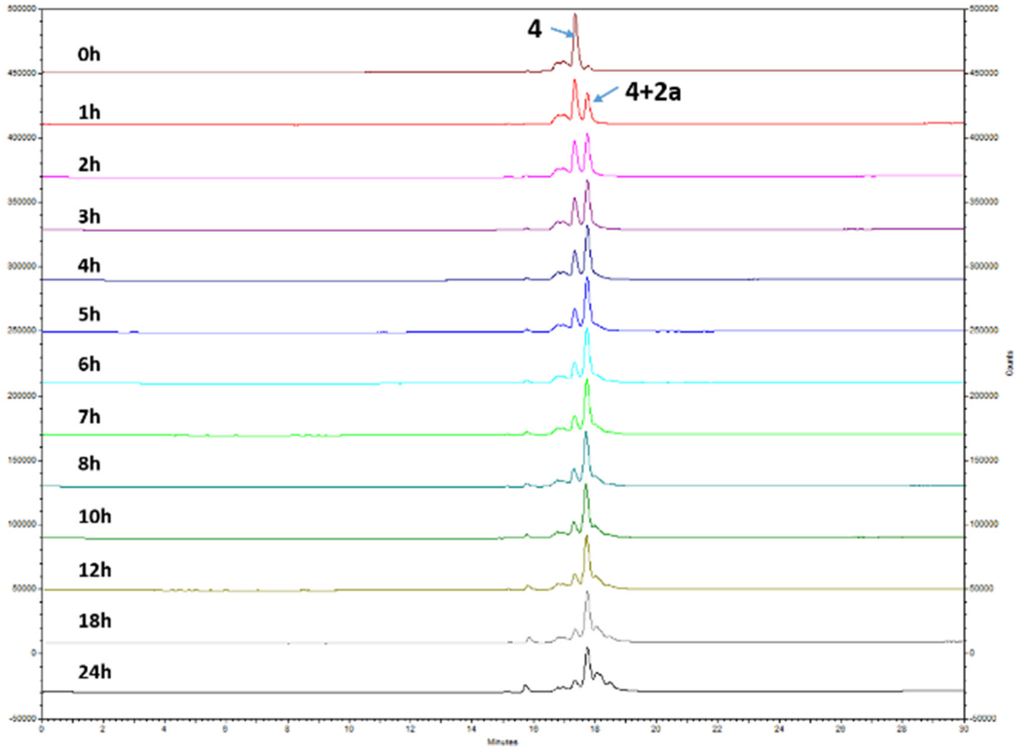

Figure S16a. Representative HPLC chromatograms (fluorescence) of the PEGylation of **4** with **2a** (0.35 kDa).

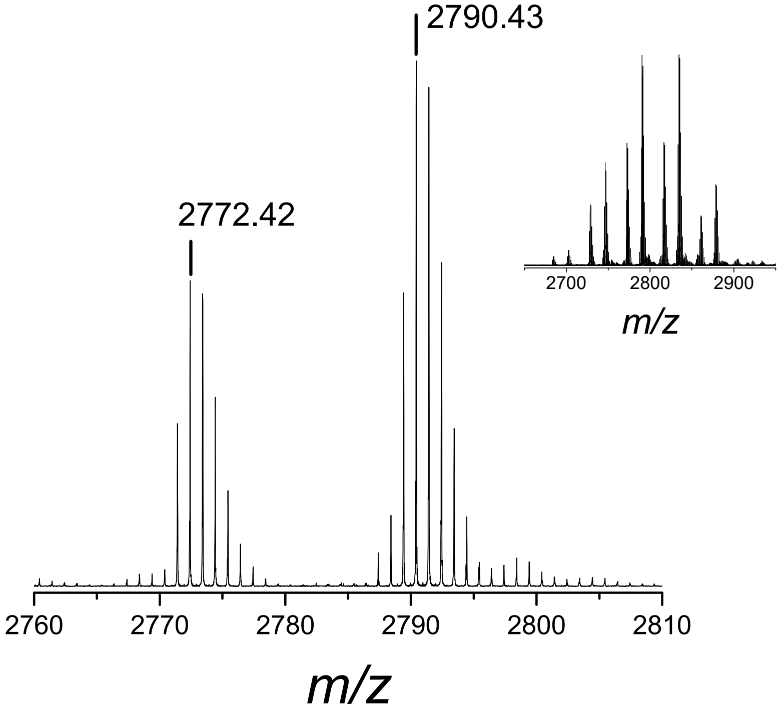

Figure S16b. Mass spectrum of [**4**+**2a**] (0.35 kDa). HRMS (ESI-ion trap) m/z: Parent ions: $[M + H]^{1+}$ Calcd for $C_{135}H_{198}N_{27}O_{35}S^{+}$ 2790.43 (Found: 2790.43); $[M – H_2O + H]^{+}$ Calcd for $C_{135}H_{196}N_{27}O_{34}S^{+}$ 2772.42 (Found: 2772.42).

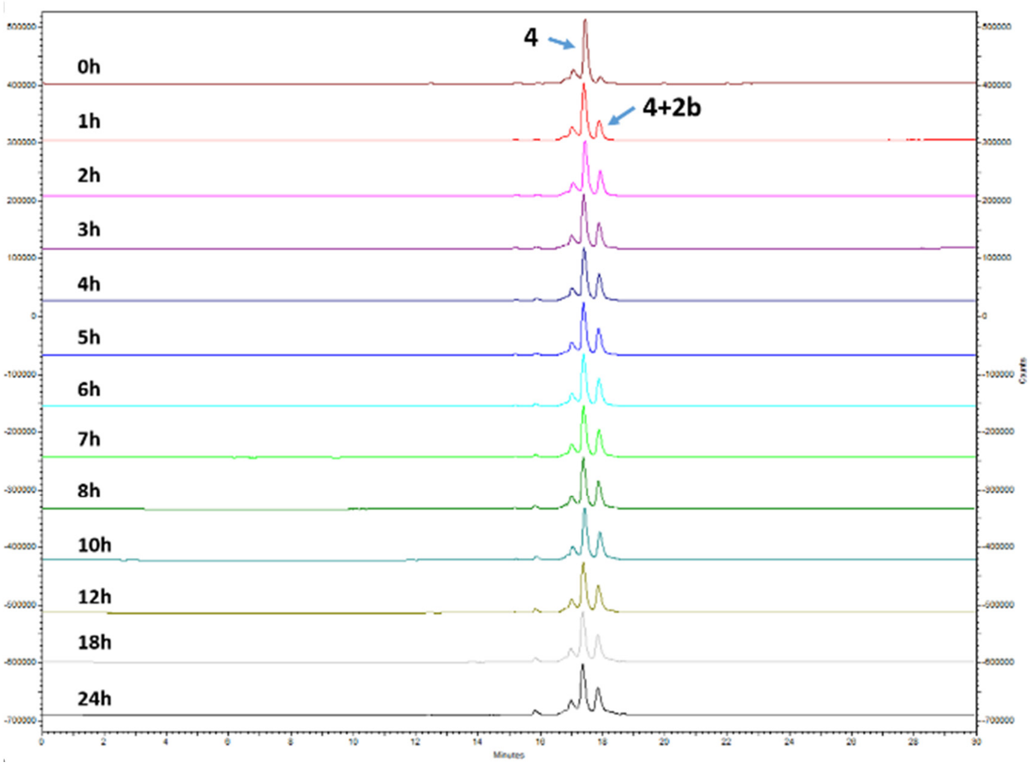

Figure S17a. Representative HPLC chromatograms (fluorescence) of the PEGylation of **4** with **2b** (0.35 kDa).

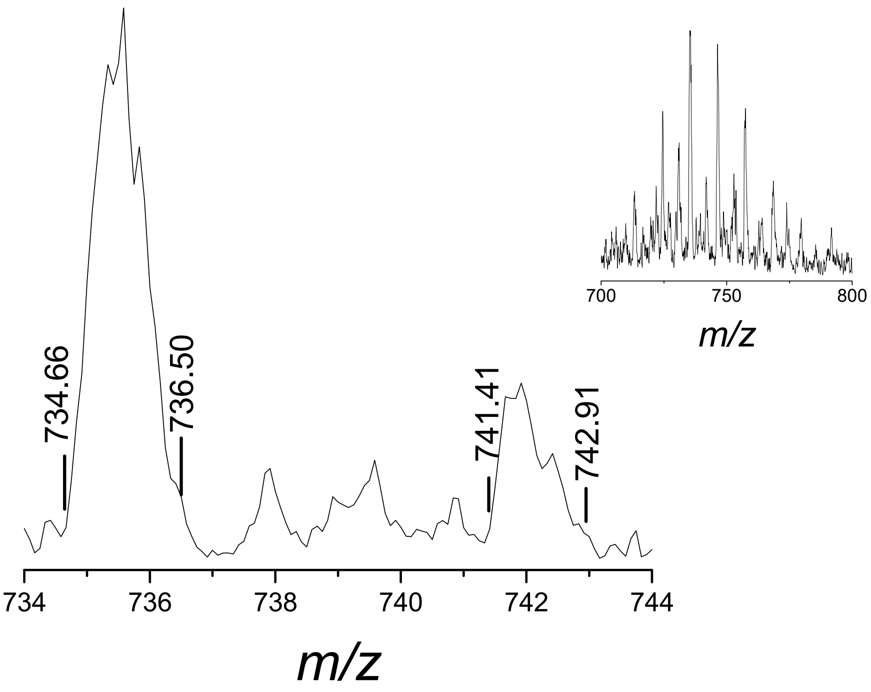

Figure S17b. Mass spectrum of [**4**+**2b**] (0.35 kDa). HRMS (ESI-ion trap) m/z: Parent ions: [M + 4H]$^{4+}$ Calcd isotope distribution for ¼ × $C_{142}H_{215}N_{27}O_{38}S^{4+}$ 734.63–736.13 (Found: 734.66–736.50); [M – H_2O + 4H]$^{4+}$ Calcd isotope distribution for ¼ × $C_{144}H_{217}N_{27}O_{38}S^{4+}$ 741.14 – 742.64 (Found: 741.41–742.91).

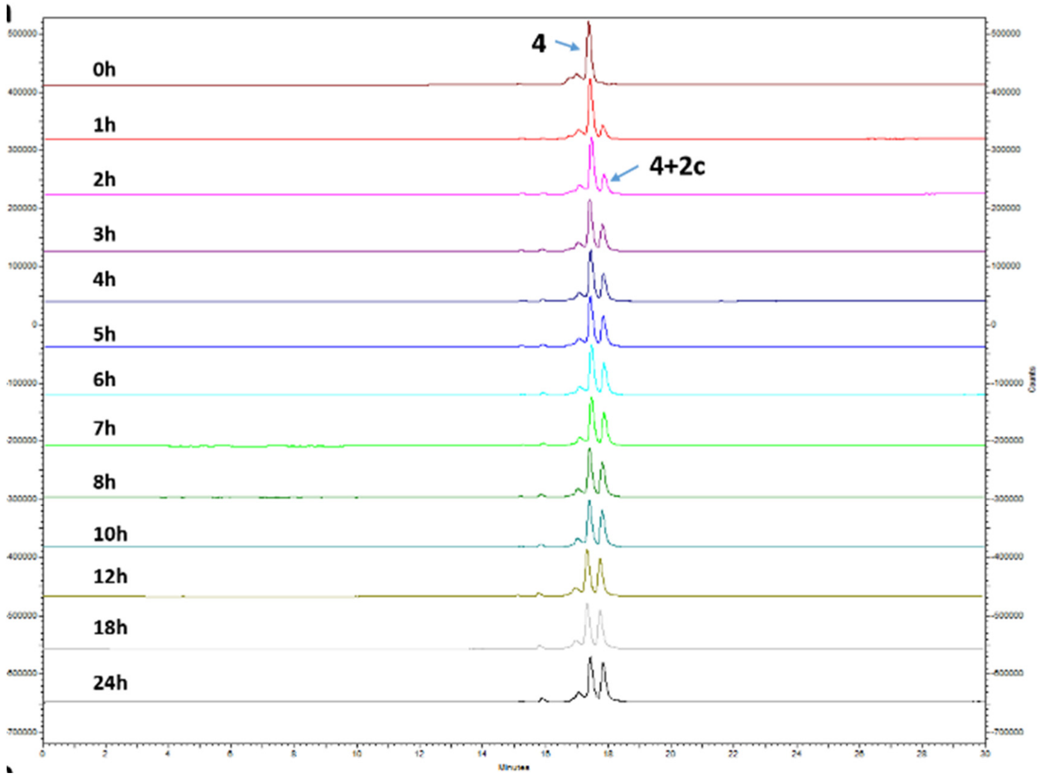

Figure S18a. Representative HPLC chromatograms (fluorescence) of the PEGylation of **4** with **2c** (0.35 kDa).

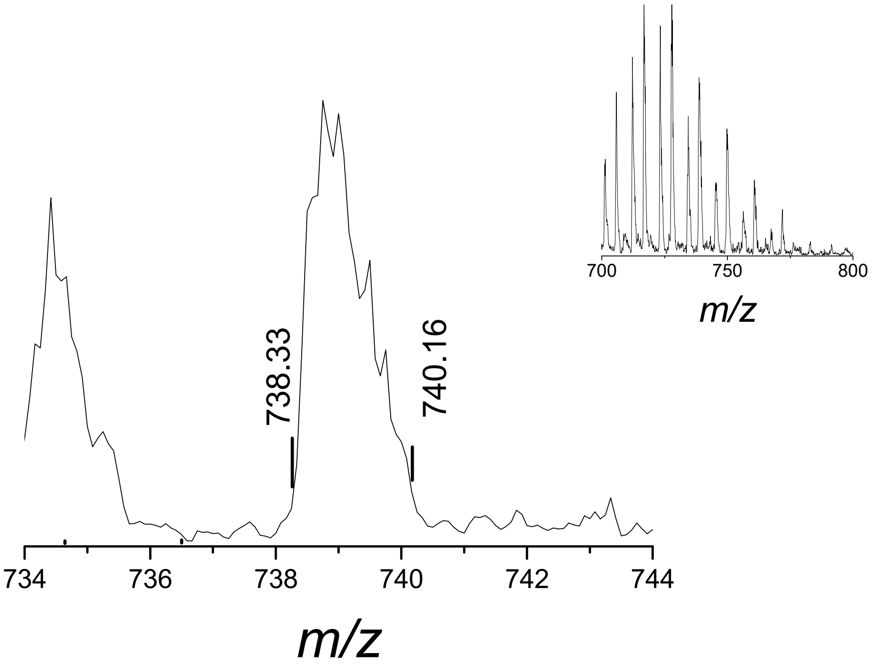

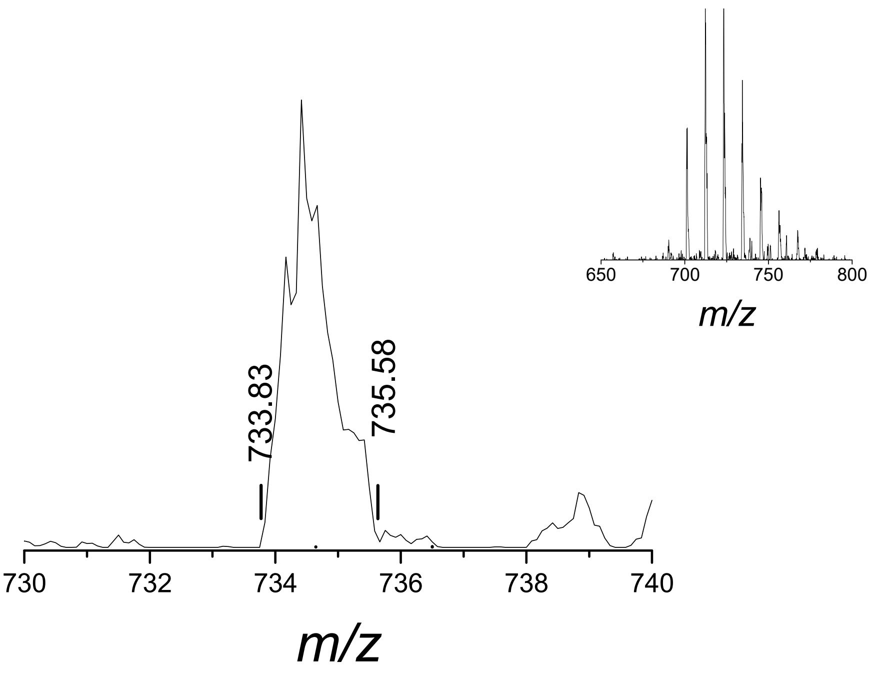

Figure S18b. Mass spectrum of [**4**+**2c**] (0.35 kDa). HRMS (ESI-ion trap) m/z: Parent ions: [M + 4H]$^{4+}$ Calcd isotope distribution for ¼ × $C_{142}H_{215}N_{27}O_{39}S^{4+}$ 738.63–740.14 (Found: 738.33–740.16); [M –H_2O + 4H]$^{4+}$ Calcd isotope distribution for ¼ × $C_{142}H_{213}N_{27}O_{38}S^{4+}$ 734.13–735.63 (Found: 733.83–735.58)

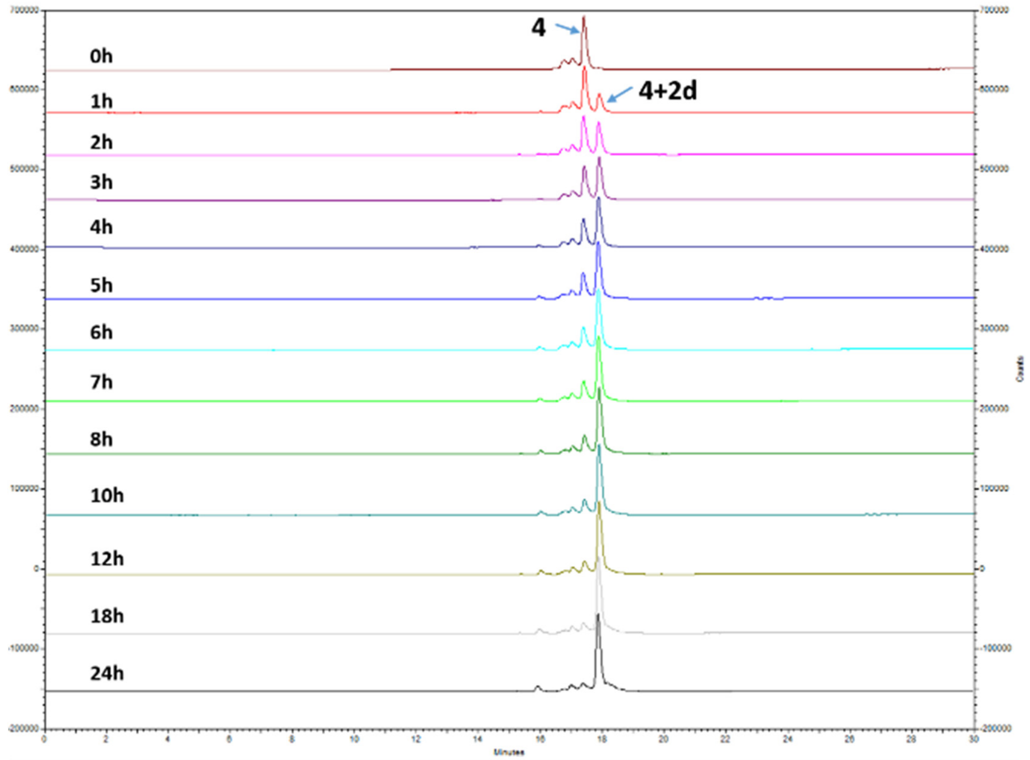

Figure S19a. Representative HPLC chromatograms (fluorescence) of the PEGylation of **4** with **2d** (0.35 kDa).

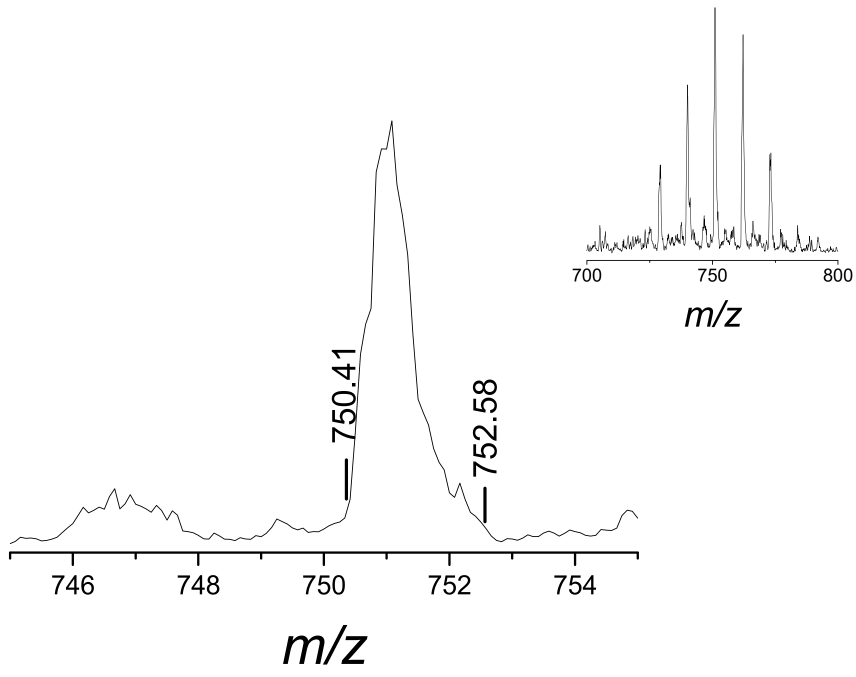

Figure S19b. Mass spectrum of [**4**+**2d**] (0.35 kDa). HRMS (ESI-ion trap) m/z: Parent ions: $[M + 4H]^{4+}$ Calcd isotope distribution for ¼ × $C_{143}H_{216}ClN_{27}O_{39}S^{4+}$ 750.63–752.63 (Found: 750.41–752.58).

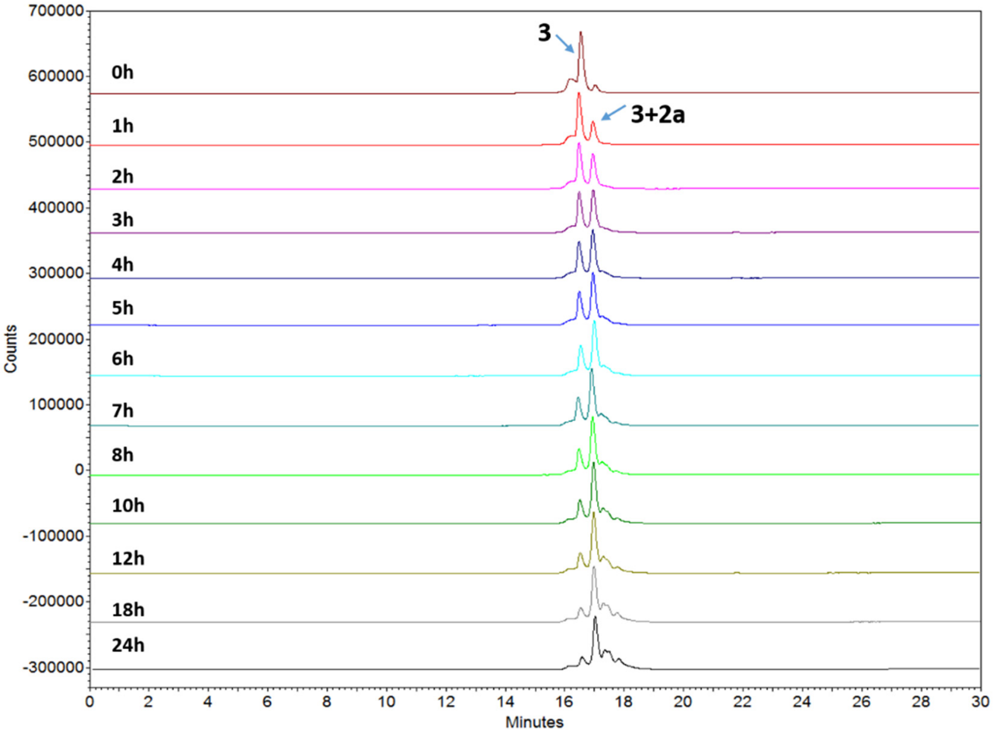

Figure S20. Representative HPLC chromatograms (fluorescence) of the PEGylation of **3** with **2a** (0.35 kDa).

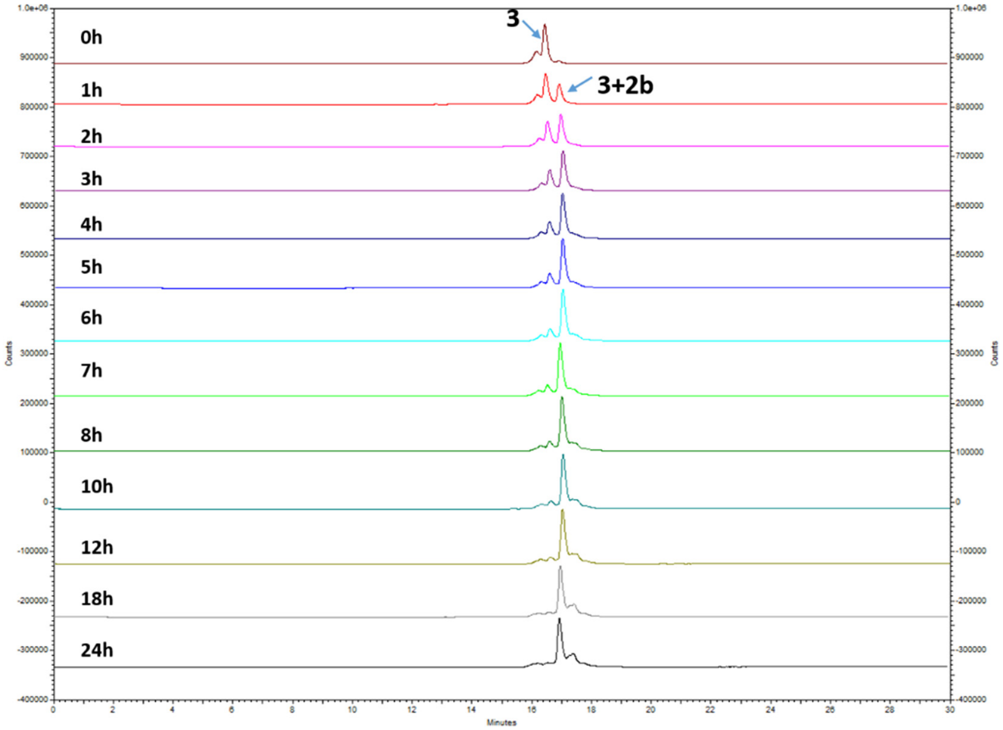

Figure S21. Representative HPLC chromatograms (fluorescence) of the PEGylation of **3** with **2b** (0.35 kDa).

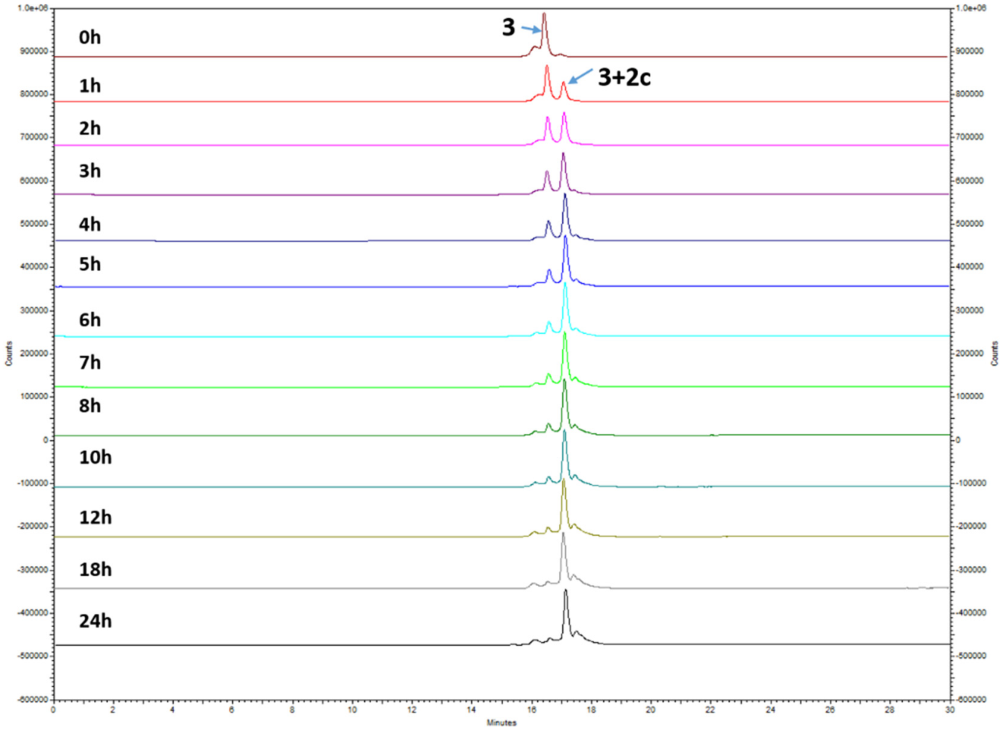

Figure S22. Representative HPLC chromatograms (fluorescence) of the PEGylation of **3** with **2c** (0.35 kDa).

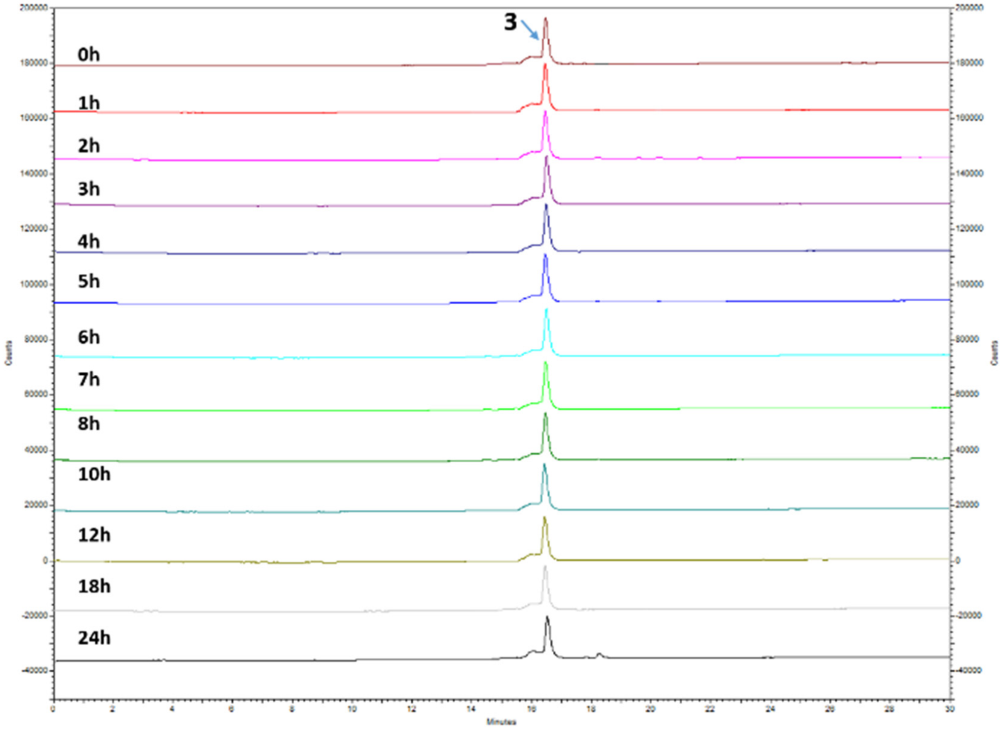

Figure S23. Representative HPLC chromatograms (fluorescence) of the PEGylation of **3** with **2d** (0.35 kDa).

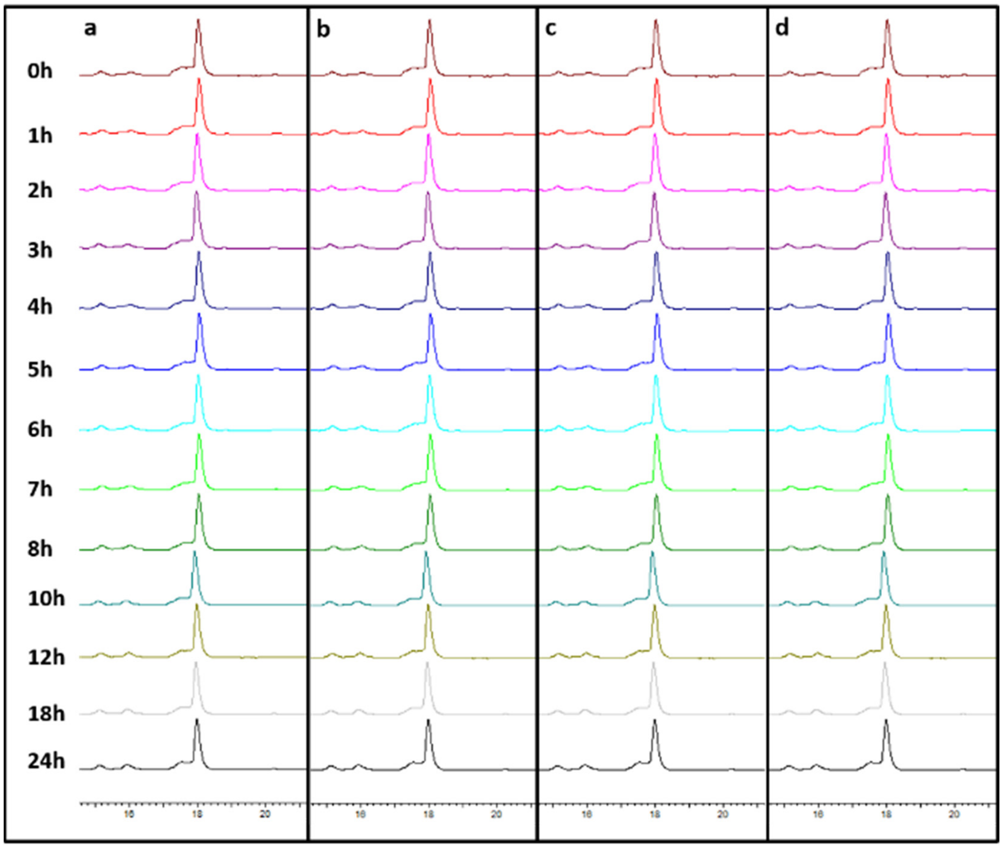

Figure S24. Representative HPLC chromatograms (fluorescence) of the PEGylation process of **5** with **2a** (a), **2d** (b), **2c** (c), or **2b** (d) (0.35 kDa). Because **5** does not possess arginine residues, no evolution of the chromatograms is observed.

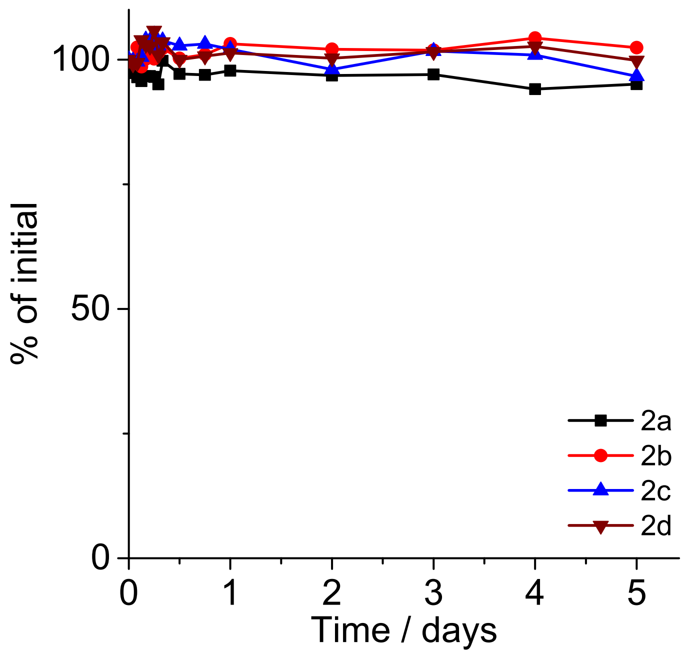

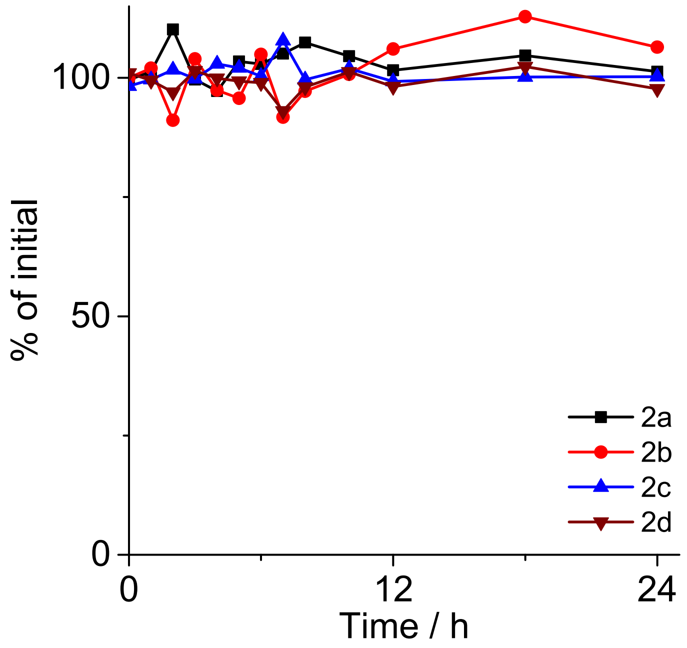

Figure S25. Reaction of GKGKGW (top) or GHGSGYG (bottom) with 4 eq. of **2a–d** (0.35 kDa) in 100 mM phosphate buffer pH 7.4 (n = 1).

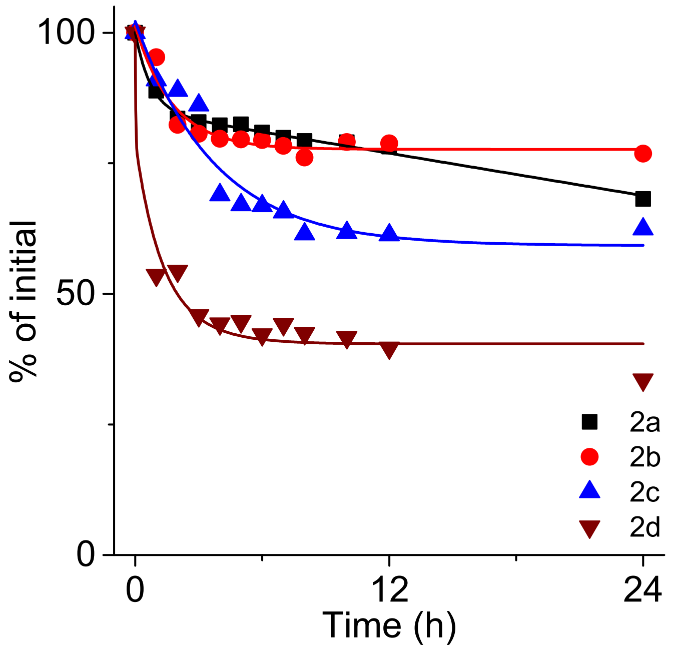

Figure S26. Reaction of cysteine with 1 eq. of **2a–d** (0.35 kDa) in 100 mM phosphate buffer pH 7.4 over 24 h (n = 1). These reactions are incomplete and plateau because of the oxidation of cysteine to cystine.

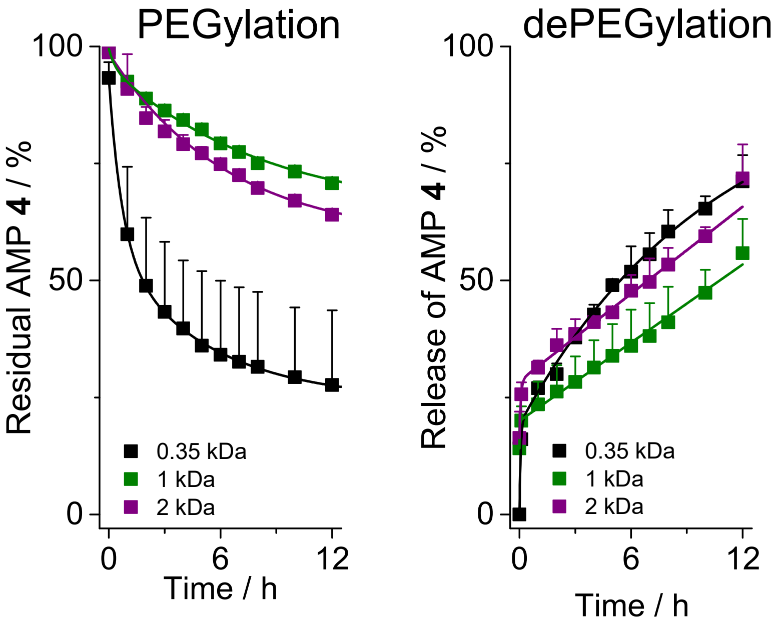

Figure S27. Influence of mPEG molecular weight on PEGylation and dePEGylation of AMP **4** with **2a**. Increasing mPEG molecular weight decreases the rate of reaction, though only small differences were observed between 1 and 2 kDa. mPEG molecular weight only slightly influenced the rate of release (given by the slope), within the range of molecular weights tested. Data presented as Mean + SD (n = 3).

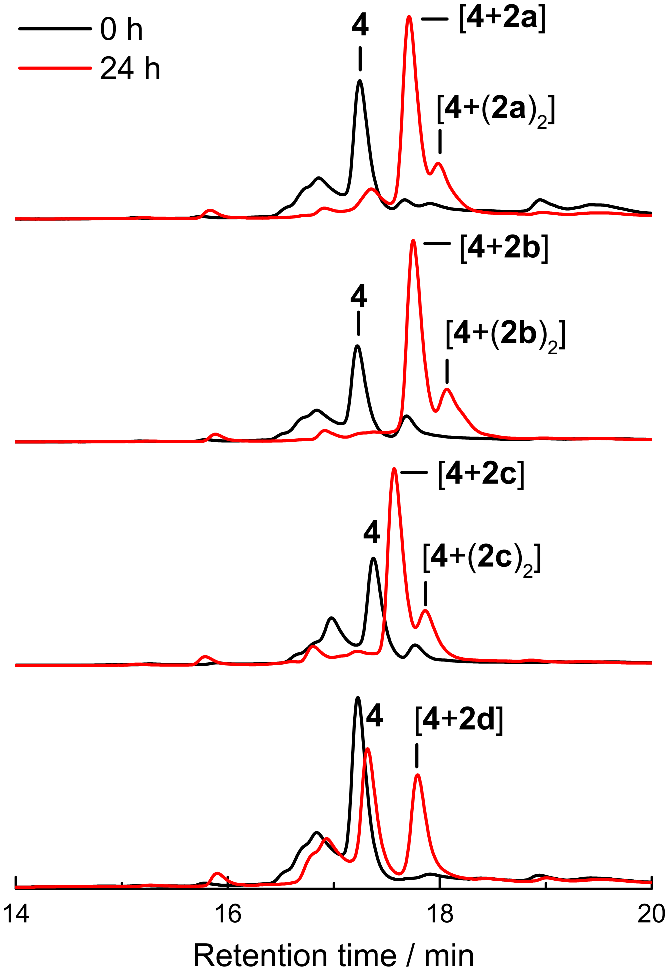

Figure S28a. PEGylation of **4** with **2a–d** in 100 mM borate buffer pH 9. Disappearance of 4 was accompanied by the appearance of one or two conjugate peaks, corresponding to the mono- or di-PEGylated conjugates. Mass spectrometry of the conjuates (Figures S28b–i) reveals only the presence of form **7** of the conjugates. Form **8** was not observed due to complexation of form **7** by borate.

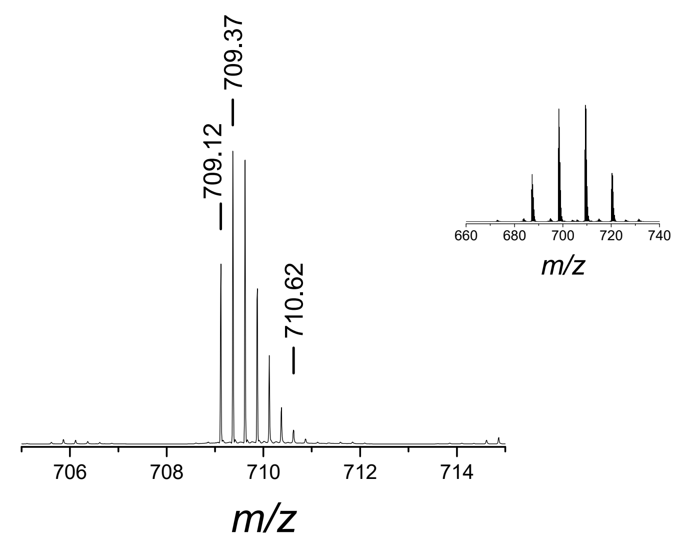

Figure S28b. Mass spectrum of [**4**+**2a**] (0.35 kDa) produced in borate buffer. HRMS (ESI-ion trap) m/z: Parent ions: $[M + 4H]^{4+}$ Calcd for ¼ × $C_{137}H_{205}N_{27}O_{36}S^{4+}$ 709.37 (Found: 709.37).

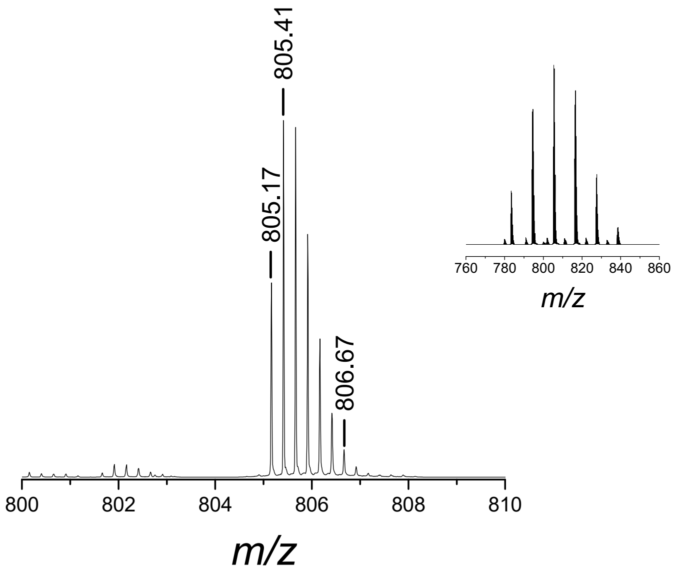

Figure S28c. Mass spectrum of [**4**+(**2a**)$_2$] (0.35 kDa) produced in borate buffer. HRMS (ESI-ion trap) m/z: Parent ions: $[M + 4H]^{4+}$ Calcd for ¼ × $C_{156}H_{233}N_{27}O_{44}S^{4+}$ 805.41 (Found: 805.41).

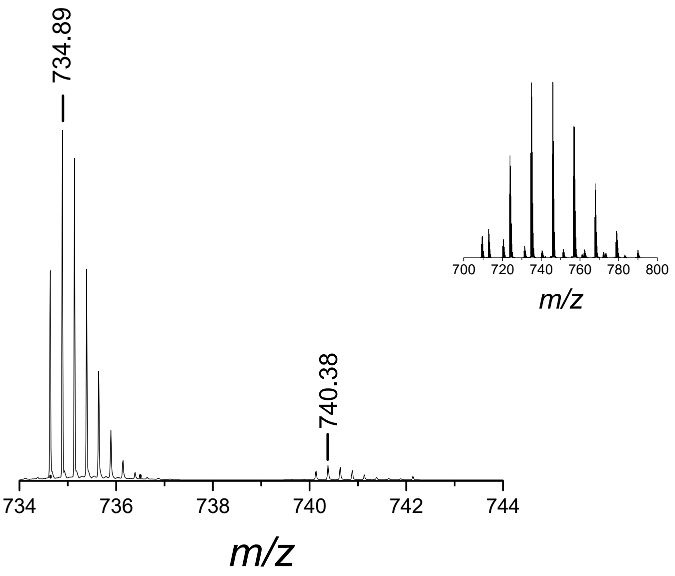

Figure S28d. Mass spectrum of [**4**+**2b**] (0.35 kDa) produced in borate buffer. HRMS (ESI-ion trap) m/z: Parent ions: $[M + 4H]^{4+}$ Calcd for ¼ × $C_{142}H_{215}N_{27}O_{38}S^{4+}$ 734.88 (Found: 734.89); $[M + 3H + Na]^{4+}$ Calcd for ¼ × $C_{140}H_{210}N_{27}NaO_{38}S^{4+}$ 740.38 (Found: 740.38).

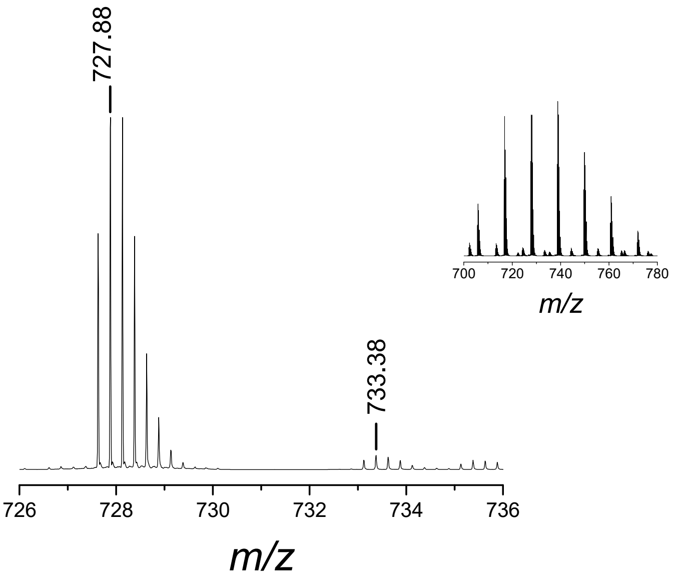

Figure 28e. Mass spectrum of [**4**+**2c**] (0.35 kDa) produced in borate buffer. . HRMS (ESI-ion trap) m/z: Parent ions: $[M + 4H]^{4+}$ Calcd for ¼ × $C_{140}H_{211}N_{27}O_{38}S^{4+}$ 727.88 (Found: 727.88); $[M + 3H + Na]^{4+}$ Calcd for ¼ × $C_{142}H_{214}N_{27}NaO_{38}S^{4+}$ 733.37 (Found: 733.38).

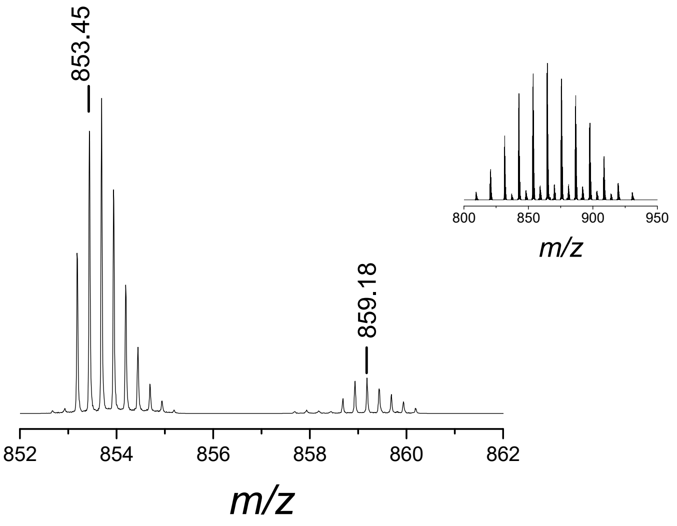

Figure 28f. Mass spectrum of [**4**+**(2c)$_2$**] (0.35 kDa) produced in borate buffer. . HRMS (ESI-ion trap) m/z: Parent ions: $[M + 4H]^{4+}$ Calcd for ¼ × $C_{164}H_{249}N_{27}O_{49}S^{4+}$ 853.44 (Found: 853.45); $[M + 3H + Na]^{4+}$ Calcd for ¼ × $C_{164}H_{248}N_{27}NaO_{49}S^{4+}$ 859.18 (Found: 859.18).

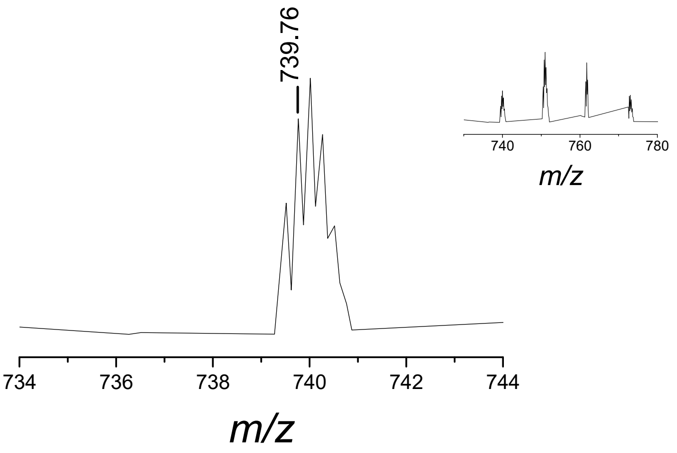

Figure 28g. Mass spectrum of [**4**+**2d**] (0.35 kDa) produced in borate buffer. HRMS (ESI-ion trap) m/z: Parent ions: $[M + 4H]^{4+}$ Calcd for ¼ × $C_{141}H_{212}ClN_{27}O_{38}S^{4+}$ 739.87 (Found: 739.76).

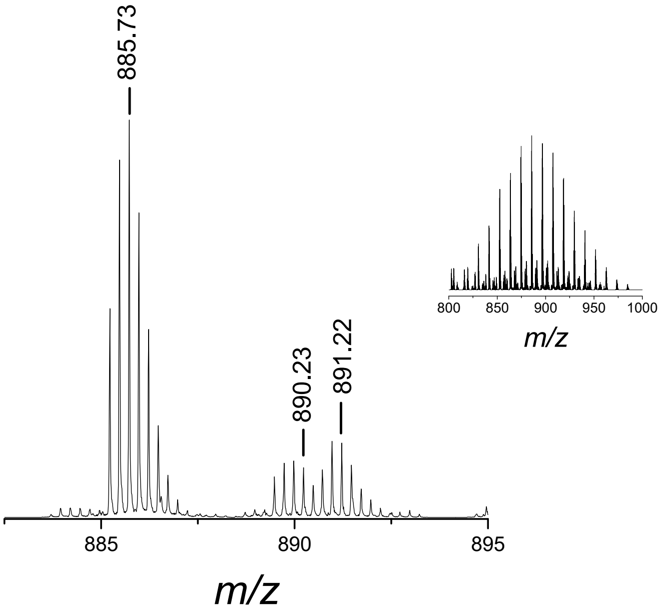

Figure 28h. Mass spectrum of [**4**+**2a**] (1 kDa) produced in borate buffer. HRMS (ESI-ion trap) m/z: Parent ions: $[M + 4H]^{4+}$ Calcd for ¼ × $C_{169}H_{269}N_{27}O_{52}S^{4+}$ 885.72 (Found: 885.73); $[M + 3H + Na]^{4+}$ Calcd for ¼ × $C_{169}H_{268}N_{27}NaO_{52}S^{4+}$ 891.22 (Found: 891.22); $[M + 4H + H_2O]^{4+}$ Calcd for ¼ × $C_{169}H_{271}N_{27}O_{53}S^{4+}$ 890.22 (Found: 890.23).

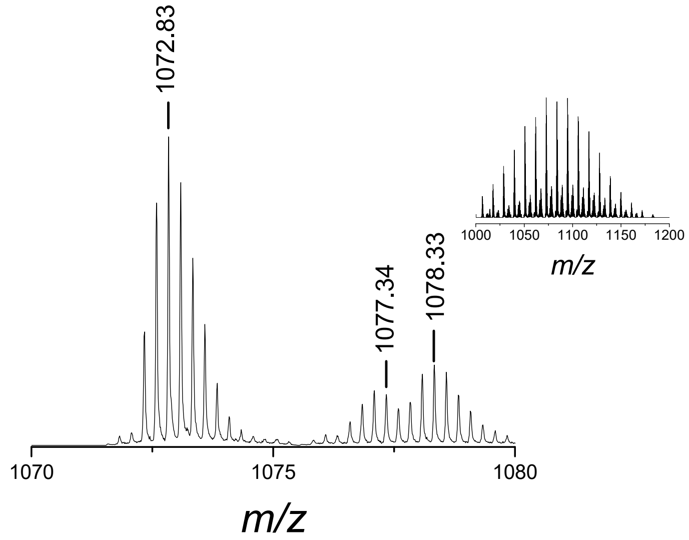

Figure 28i. Mass spectrum of [**4**+**2a**] (2 kDa) produced in borate buffer. HRMS (ESI-ion trap) m/z: Parent ions: $[M + 4H]^{4+}$ Calcd for ¼ × $C_{203}H_{337}N_{27}O_{69}S^{4+}$ 1072.84 (Found: 1072.83); $[M + 3H + Na]^{4+}$ Calcd for ¼ × $C_{203}H_{336}N_{27}NaO_{69}S^{4+}$ 1078.33 (Found: 1078.33); $[M + 4H + H_2O]^{4+}$ Calcd for ¼ × $C_{203}H_{339}N_{27}O_{70}S^{4+}$ 1077.34 (Found: 1077.34).

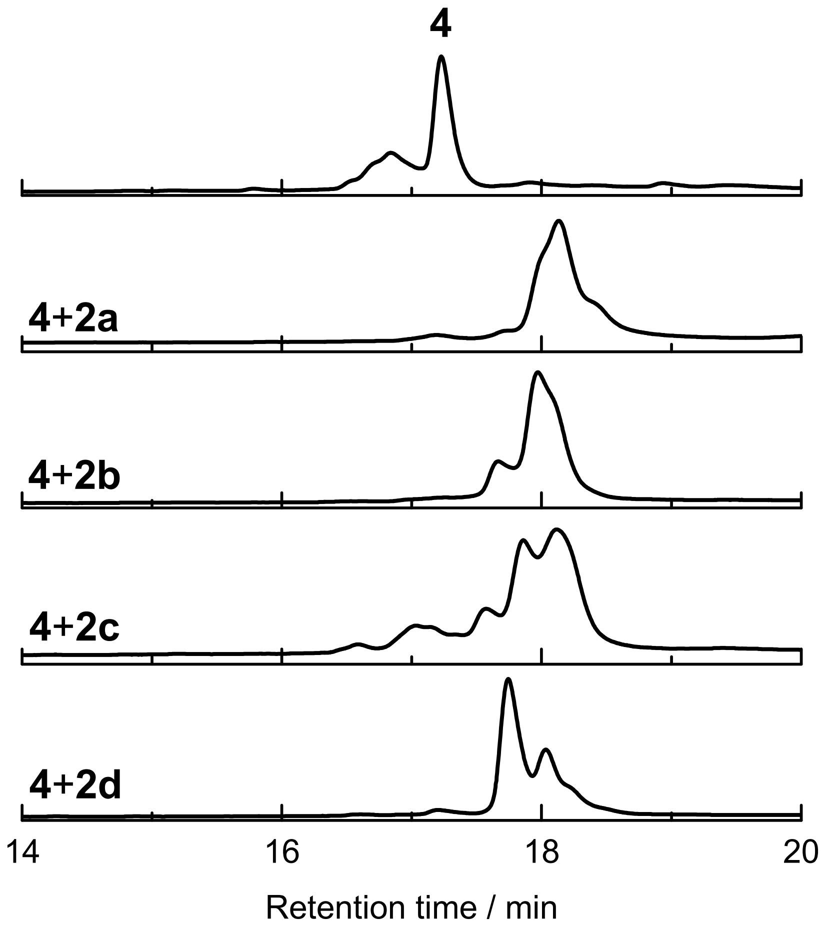

Figure S29. Stability of AMP–polymer conjugates in the freeze-dried state. Conjugates of **4** and **2a–d** were prepared in borate buffer to uniquely obtain form **7** of the conjugate. After isolation by preparative HPLC and recovery by lyophilization, the conjugates were stored at –20 ºC for 1 week. Reconstitution in 100 mM phosphate buffer (pH 4) and immediate analysis by HPLC revealed the absence of unconjugated **4** (top chromatogram).

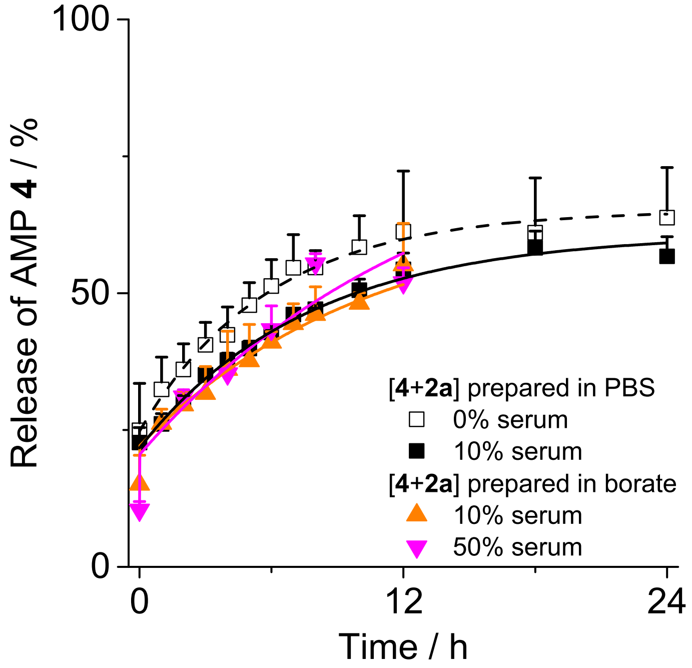

Figure S30. Influence of serum content on the dePEGylation of [**4**+**2a**] (0.35 kDa) prepared in PBS or in borate buffer. Serum (0–50%) has little or no effect on the release, based on the slopes of the curves. Conjugates prepared in borate showed lesser burst release, resulting in a small offset between the different curves. Data presented as mean + SD (n = 3).

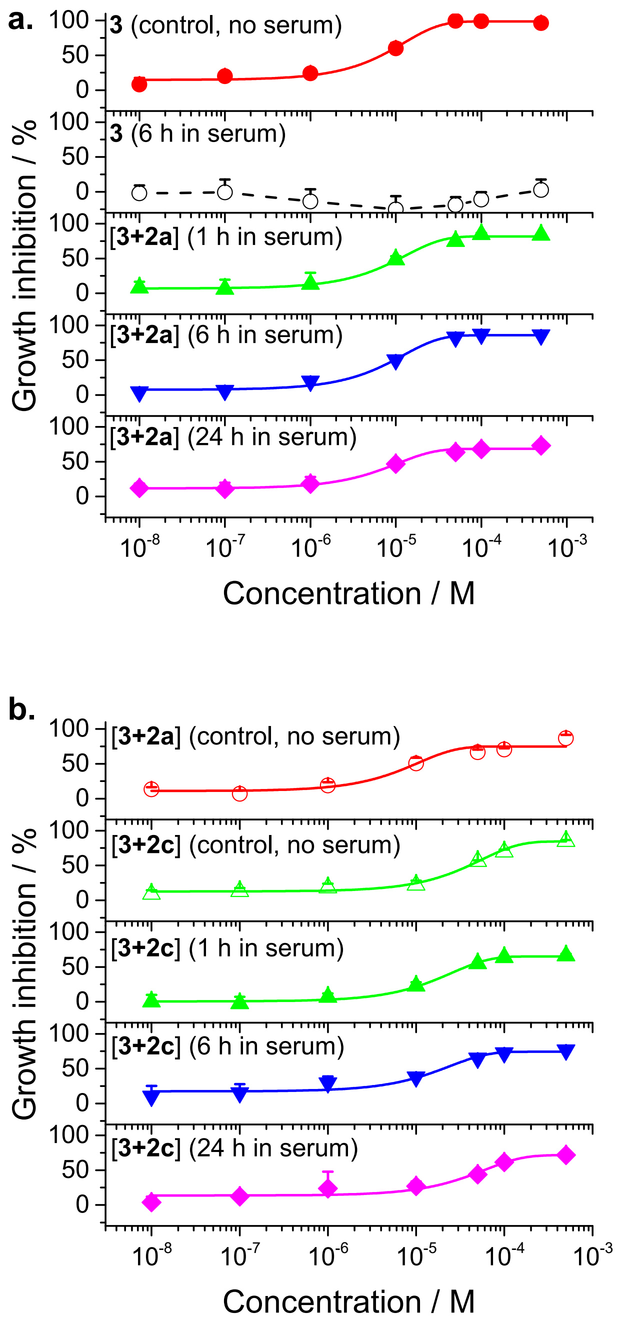

Figure S31. Dose-response curves to detemiend the antibacterial properties of **3**, and [**3+2a,c**] conjugates following exposure to trypsin-spiked serum. Data presented as mean + SD (n = 3–4).

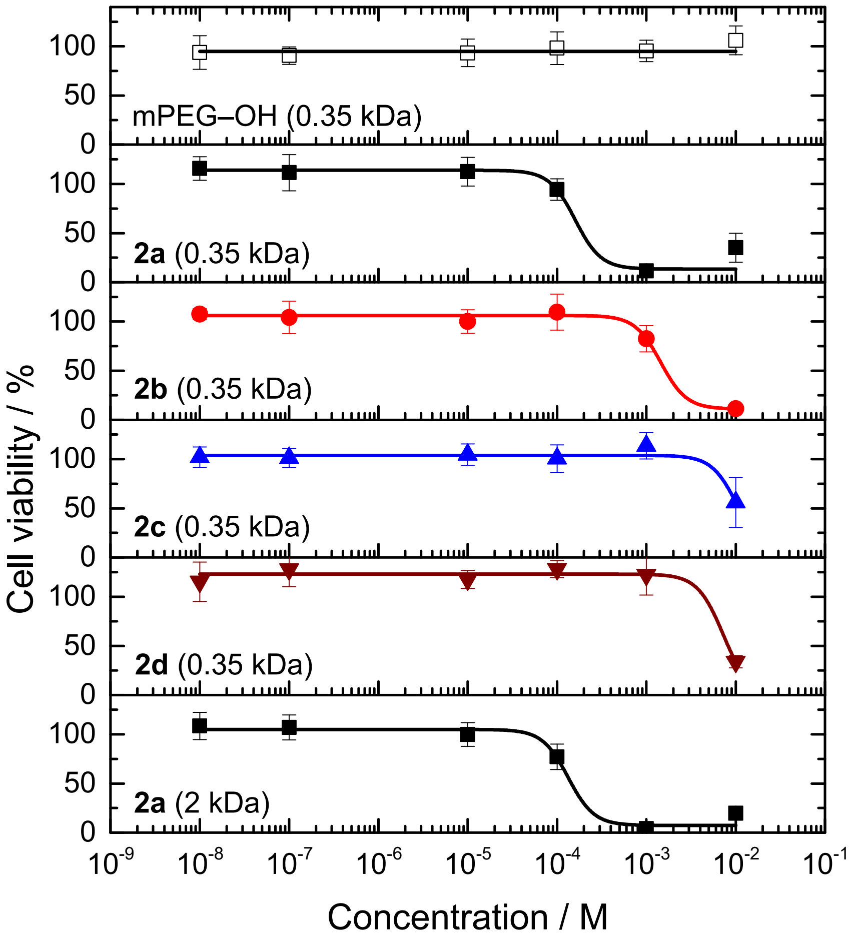

Figure S32. Evaluation of cytotoxicity of **2a–d** towards HepG2 cells. Data presented as mean ± SD (n = 9). EC_{50} values determined from Logistic fit of the experimental data using Microcal Origin. mPEG: >10 mM; **2a** (0.35 kDa): 0.16 ± 0.04 mM; **2b** (0.35 kDa): 1.4 ±0.1 mM; **2c** (0.35 kDa): 10 ± 1 mM; **2d** (0.35 kDa): 7.2 ± 0.3 mM; **2a** (2 kDa): 0.13 ± 0.06 mM.

6. Supporting references

Full listing of references with abbreviated author lists in the main manuscript:

[1] R. P. Dellinger, M. Levy, J. Carlet, J. Bion, M. Parker, R. Jaeschke, K. Reinhart, D. Angus, C. Brun-Buisson, R. Beale, T. Calandra, J.-F. Dhainaut, H. Gerlach, M. Harvey, J. Marini, J. Marshall, M. Ranieri, G. Ramsay, J. Sevransky, B. T. Thompson, S. Townsend, J. Vender, J. Zimmerman, J.-L. Vincent, *Intensive Care Med*. **2008**, *34*, 17-60.

[6c] Y. Shechter, M. Mironchik, A. Saul, E. Gershonov, L. Precido-Patt, K. Sasson, H. Tsubery, B. Mester, A. Kapitkovsky, S. Rubinraut, Y. Vachutinski, G. Fridkin, M. Fridkin, *Int. J. Pept. Res. Ther.* **2007**, *13*, 105-117.
